# Childhood maltreatment and adult diseases in the general population: the mediating role of smoking and overweight in a time-sequence design

**DOI:** 10.1186/s12889-025-24854-y

**Published:** 2025-10-27

**Authors:** Johanna Klinger-König, Fabian Streit, Maja P. Völker, Josef Frank, Peggy Sekula, Stefanie Jaskulski, Michael Leitzmann, Claudia Meinke-Franze, Carsten O. Schmidt, Thomas Keil, Stefan N. Willich, Tobias Pischon, Ilais M. Velásquez, Jonas Frost, Börge Schmidt, Jana-Kristin Heise, Carolina J. Klett-Tammen, Lena Koch-Gallenkamp, Nadia Obi, Volker Harth, Ute Mons, Klaus Berger, Karin H. Greiser, Rafael Mikolajczyk, Matthias B. Schulze, Hans J. Grabe

**Affiliations:** 1 Department of Psychiatry and Psychotherapy, University Medicine Greifswald, Ellernholzstraße 1-2, Greifswald, D-17489 Germany; 2 Department of Psychiatry and Psychotherapy, Central Institute of Mental Health, Medical Faculty Mannheim, Heidelberg University, Mannheim, Germany; 3 Hector Institute for Artificial Intelligence in Psychiatry, Central Institute of Mental Health, Medical Faculty Mannheim, Heidelberg University, Mannheim, Germany; 4 German Center for Mental Health (DZPG), Partner Site Mannheim - Heidelberg - Ulm, Mannheim, Germany; 5 Department of Genetic Epidemiology in Psychiatry, Central Institute of Mental Health, Medical Faculty Mannheim, Heidelberg University, Mannheim, Germany; 6 Institute of Genetic Epidemiology, Faculty of Medicine and Medical Center, University of Freiburg, Freiburg, Germany; 7 Institute for Prevention and Cancer Epidemiology, Faculty of Medicine and Medical Center, University of Freiburg, Freiburg, Germany; 8 Institute for Epidemiology and Preventive Medicine, University of Regensburg, Regensburg, Germany; 9 Institute for Community Medicine, University Medicine Greifswald, Greifswald, Germany; 10 Institute of Social Medicine, Epidemiology and Health Economics, Charité – Universitätsmedizin Berlin, Berlin, Germany; 11 Institute for Clinical Epidemiology and Biometry, University Würzburg, Würzburg, Germany; 12 Landesinstitut Gesundheit I, Bavarian Health and Food Safety Authority, Erlangen, Germany; 13 Molecular Epidemiology Research Group, Max Delbrück Center for Molecular Medicine in the Helmholtz Association (MDC), Berlin, Germany; 14 Charité - Universitätsmedizin Berlin, corporate member of Freie Universität Berlin and Humboldt-Universität zu Berlin, Berlin, Germany; 15 Institute for Medical Epidemiology, Biometrics, and Informatics, Medical Faculty of the Martin Luther University Halle-Wittenberg, Halle, Germany; 16 Institute for Medical Informatics, Biometry and Epidemiology, University Hospital of Essen, University of Duisburg-Essen, Essen, Germany; 17 Department of Epidemiology, Helmholtz Centre for Infection Research, Brunswick, Germany; 18 Division of Clinical Epidemiology and Aging Research, German Cancer Research Center (DKFZ), Heidelberg, Germany; 19 Institute for Occupational and Maritime Medicine (ZfAM), University Medical Center Hamburg-Eppendorf, Hamburg, Germany; 20 Division Primary Cancer Prevention, German Cancer Research Center (DKFZ), Heidelberg, Germany; 21 Medical Faculty Mannheim, Heidelberg University, Mannheim, Germany; 22 Institute of Epidemiology and Social Medicine, University of Münster, Münster, Germany; 23 Division of Cancer Epidemiology, German Cancer Research Center (DKFZ), Heidelberg, Germany; 24 Department of Molecular Epidemiology, German Institute of Human Nutrition Potsdam-Rehbruecke, Nuthetal, Germany; 25 Institute of Nutritional Science, University of Potsdam, Nuthetal, Germany; 26 German Center for Neurodegenerative Diseases (DZNE), Partner Site Rostock/Greifswald, Greifswald, Germany

**Keywords:** Childhood abuse, Childhood neglect, Age of onset, Mental health, Chronic non-communicable diseases

## Abstract

**Background:**

Childhood maltreatment is associated with an unhealthier lifestyle in adulthood and an increased risk of mental and somatic health problems, although the underlying pathways remain unclear. This study examined whether smoking and overweight mediate the association between childhood abuse/neglect and frequent adult diseases, including cancer, myocardial infarction, stroke, type 2 diabetes, chronic obstructive pulmonary disease, depression, and anxiety.

**Methods:**

Childhood maltreatment was assessed in 152,887 German National Cohort (NAKO) participants using the Childhood Trauma Screener. Information on smoking initiation age, weight history, and respective age at diagnosis was incorporated to ensure that smoking and overweight preceded the diagnosis. Mediation analyses were adjusted for age, sex, study center, and education.

**Results:**

For childhood abuse, larger proportions of associations with adult somatic diseases were mediated through preexisting smoking and overweight compared to adult mental disorders. Smoking most strongly mediated myocardial infarction (36.88% [95% confidence interval (CI): 17.88%; 55.89%]), with more pronounced effects in men (48.62% [14.28%; 82.97%]) than in women (20.82% [2.75%; 38.89%]). For overweight, a substantial mediation was only found for type 2 diabetes (13.69% [9.85%; 17.52%]), with stronger effects in women (16.16% [8.92%; 23.39%]) compared to men (8.43% [4.52%; 12.35%]). Comparable results were found for childhood neglect.

**Conclusions:**

To smoke or be overweight before the first diagnosis of myocardial infarction and type 2 diabetes mediated the association between childhood abuse/neglect and these somatic diseases. However, while the mediation through smoking and overweight contributed to the disease risk linked to childhood maltreatment, strong direct effects of childhood abuse/neglect persisted for both mental and somatic health problems. These findings underscore the need for further longitudinal studies to better understand the pathways.

**Supplementary Information:**

The online version contains supplementary material available at 10.1186/s12889-025-24854-y.

## Background

Childhood maltreatment, comprising emotional, physical, or sexual abuse, and emotional or physical neglect, is reported by 20–33% of the German adult population [1–3]. It is associated with an unhealthier lifestyle and exacerbates mental and somatic health problems [4–7].

Various mental disorders have been associated with childhood maltreatment, with the most consistent findings for depressive and anxiety disorders [4, 6, 8]. Besides increased psychological morbidity, childhood maltreatment has also been linked to a younger age of onset, more severe disease courses, reduced treatment responsiveness, and more frequent comorbidities [4, 8, 9].

Although studied less frequently, childhood maltreatment was also associated with increased somatic morbidity, including type 2 diabetes, myocardial infarction (MI), stroke, and cancer [4, 6, 10, 11]. Similar to findings for mental disorders, this association was more pronounced in women than men and younger compared to older adults [7, 12].

Hildyard and Wolfe [13] described physical and sexual abuse as a more intense, event-specific type of childhood maltreatment, whereas they considered physical neglect as a more chronic type where individual events are difficult to delineate. In several studies, stronger associations with health issues in adulthood were described for abuse compared to neglect. Thus, associations with depression were stronger for abuse than neglect, and chronic neglect was more closely related to depression than single episodes [14, 15]. Additionally, associations between mental disorders and abuse were more strongly influenced by behavioral risk factors, such as smoking and obesity, compared to associations with neglect [15]. In contrast, diabetes was primarily associated with neglect [16].

The exact pathways between childhood maltreatment and mental or somatic diseases are still not fully understood. Unhealthy lifestyles, such as smoking, obesity, and alcohol consumption, are discussed [17–19]. Indeed, adjusting for unhealthy lifestyles attenuated the associations between adverse childhood experiences, including maltreatment, and depression, cardiovascular diseases (CVD), chronic obstructive pulmonary disease (COPD), and diabetes [20–23]. However, most studies assessed mediators and outcomes simultaneously, complicating the establishment of time sequences and leading to partly contradictory results between cross-sectional and longitudinal studies [5, 20–26].

Moreover, lifestyle factors and diseases may interact bidirectionally. Smoking and unhealthy diets may serve as coping mechanisms for emotional distress and mental health challenges [27–29]. In contrast, cancer, MI, stroke, diabetes, and COPD often lead to smoking cessation [30, 31]. As part of the disease course, depression, anxiety, and breast cancer were linked to weight gain, particularly in women [32–34], whereas COPD was associated with weight loss [35, 36].

Hence, establishing an accurate time sequence between mediators and outcomes is crucial for evaluating mediation effects between childhood maltreatment and adult health. Although pathways might differ between abuse and neglect, previous research either focused on one specific type of maltreatment or neglected these differences [14–17]. Clarifying these pathways is relevant because it distinguishes the contribution of childhood maltreatment from that operating through modifiable factors, thereby identifying targets for prevention and intervention later in life. The present study used data from a large population-based cohort with a clear time sequence between mediators and outcomes to address this issue. The study aimed to, first, investigate whether smoking or overweight mediates associations between abuse and neglect and seven diseases [7]. Second, we estimated sex- and age-differences in these associations. Third, we examined whether abuse and neglect influenced the age at diagnosis.

## Methods

The German National Cohort (NAKO) is a multi-center, general population cohort that examined 205,415 participants in 18 study centers across 13 out of 16 German federal states at baseline [37]. NAKO aims to understand common disease causes, identify risk factors, and improve early detection and prevention strategies. Ethics approval was obtained from all study centers. All participants gave written informed consent. Assessments and analyses followed the Declaration of Helsinki.

### Data assessment

During standardized interviews, trained study nurses collected sociodemographic data, including age, sex (men/women), and education. Educational years were categorized according to the International Standard Classification of Education 97 (ISCED-97) [38].

Additionally, participants were asked if they had ever been diagnosed with different disorders, including:

- Any cancer.
- Myocardial infarction (hereafter: MI).
- Stroke.
- Diabetes.
- Chronic bronchitis or chronic obstructive pulmonary disease (hereafter: COPD).
- Anxiety disorder or panic attacks (hereafter: anxiety).
- Any depressive disorder (hereafter: depression).

Affected participants were asked for their age at first diagnosis. Participants were excluded if they did not report any age at diagnosis or if they were diagnosed before the age of 18 years to prevent potential feedback between childhood illness and childhood maltreatment [6]. Based on the reported type of the first tumor, any cancer was distinguished into smoking-related (oral cavity, pharynx, esophagus, stomach, pancreas, intestines, larynx, lung, kidney, urinary tract, urinary bladder, leukemia, liver, breast [women], cervix) [39–42] and obesity-related (oral cavity, pharynx, esophagus, stomach, pancreas, intestines, larynx, skin, kidney, urinary tract, thyroid, brain, non-Hodgkin’s lymphoma, liver, gall bladder, breast, prostate, uterus, ovaries) [43–45]. Type 2 diabetes was defined according to Tanoey et al. [46], excluding type 1 diabetes and gestational diabetes.

Participants who reported that they had ever smoked were asked about their age at smoking initiation. Using this smoking initiation age and the age at diagnosis, we identified participants who smoked before they were diagnosed separately for each disease (ever smoking). The amount the participants smoked was not taken into account. For sensitivity analyses, participants who also reported to have quit smoking before the diagnosis were excluded, thus comparing never to current smokers (current smoking).

Participants reported their weight compared to peers at age 10 (lower/regular/higher) and their weights at ages 18, 30, and 50. The current weight and height measured during baseline examinations were used to calculate the BMI (kg/m²) at ages 18, 30, 50, and baseline [47]. We defined early-onset overweight as a higher weight at age 10 or a BMI ≥ 25 at age 18; late-onset overweight referred to a BMI ≥ 25 at ages 30 or 50. Analogously to smoking, we identified participants who were overweight before the diagnosis. To account for the possibility that overweight at age 10 might interfere with childhood maltreatment, we defined early-onset overweight during adulthood (18+) by excluding participants who were solely classified as early overweight based on their reports at age 10 and used this variable in sensitivity analyses.

Only smoking and weight history were assessed retrospectively, allowing the implementation of a precise time sequence between childhood maltreatment and subsequent diagnoses. Other lifestyle factors, such as alcohol consumption, physical activity, or diet, were assessed only at the time of recruitment. These factors could therefore not be temporally ordered between childhood maltreatment and the diagnosis, and were thus not the focus of the present analyses.

Childhood maltreatment was self-reported using the Childhood Trauma Screener (CTS), the five-item ultra-short version of the Childhood Trauma Questionnaire (CTQ), with high reliability and validity [48, 49]. Each item is rated on a five-point scale (1 = never to 5 = very often) and assesses one CTQ subscale: emotional, physical, and sexual abuse and emotional and physical neglect. Glaesmer et al. proposed cut-offs that mimic the categorization of the CTQ subscales into none/mild vs. moderate/severe [3, 50]. In detail, item-levels moderate/severe were defined as ≥ 3 (“sometimes” to “very often”) for emotional and physical abuse, ≥ 2 (“rarely” to “very often”) for sexual abuse, and ≤ 2 (“never” to “rarely”) for emotional and physical neglect. Note that the neglect items are positively phrased (e.g., “I felt loved.”), and thus lower ratings indicate more neglect. Using these cut-offs, childhood abuse was defined as at least one type of moderate/severe abuse (emotional/physical/sexual); moderate/severe neglect was defined as moderate/severe emotional or physical neglect. Abuse and neglect were used as separate exposures in the present analyses.

### Analytic sample

Relative to the source population, the NAKO baseline sample contained fewer participants with a migration background, with low and medium education, and fewer single-person households [51]. For the present analyses, participants without information on the CTS (*n* = 32,782), smoking (*n* = 9,672), weight history (*n* = 33,197), disease history (*n* = 289), or educational years (*n* = 18,719) were excluded, leading to an analytic sample of 152,885 participants. Compared with participants excluded from the analyses, those retained were younger and more highly educated (Table S1). Sample sizes for individual models varied disease-specifically due to missing data for each condition.

### Statistical analyses

Statistical analyses were conducted with R (*version 4.2.1*). Descriptive statistics are reported as mean (M), 25th and 75th percentiles (25%; 75%) for continuous and as percentages (%) for dichotomous variables.

First, for the individual paths between exposure, mediator, and outcome, we implemented generalized linear models (GLM) with a log link and Poisson mean with robust standard errors. These included associations between childhood maltreatment and the mediators (Path A), the mediators and the diseases (Path B), and childhood maltreatment and the diseases (Path C). Participants without abuse or neglect, never smokers, and participants without overweight served as the reference group, respectively. Differences in the mean age at diagnosis were modeled in affected participants using linear regression. Risk ratios (RR) and unstandardized regression coefficients (β) are reported with 95% confidence intervals (95%-CI).

Afterwards, mediation analyses (package: *regmedint*) [52] with an interaction term between exposure and mediator were conducted to investigate whether smoking and overweight before the diagnosis mediated the associations between abuse or neglect and the presence or age at diagnosis of the diseases. To avoid relying on the rare-outcome approximation, binary outcomes were modeled with GLMs with a log link and Poisson mean for the outcome models, yielding RR-based natural effects [53]. Binary mediators were modeled with logistic regressions [53]. For accessibility, the main manuscript reports the standard two-way decomposition: the pure direct effect (PDE), the total indirect effect (TIE), and the proportion mediated. However, because an exposure-mediator interaction was included, the PDE comprises the controlled direct effect (CDE) and the reference interaction (INT_ref_); the TIE comprises the pure natural indirect effect (PIE) and the mediated interaction (INT_med_) [54]. The full four-way decompositions are presented in Additional file 1. The interpretation focused on mediation analyses for which all three paths (A, B, and C) revealed a substantive effect, defined as RR < 0.9 or RR > 1.1 (≥ 10% risk change) or |β|>1 (≥ 1-year difference). For analyses that included the age at diagnosis, the 1-year threshold (|β|>1) captures both the recall precision of self-reported age at diagnosis and the smallest difference that reflects a measurable impact. The ± 10% risk criterion (RR < 0.9 or RR > 1.1) was chosen as the logistic criterion, ensuring sensitivity to small, yet meaningful, population effects [55, 56]. In sensitivity analyses, ever smoking and early-onset smoking were exchanged by current smoking and early-onset during adulthood (18+).

All regression and mediation models were adjusted for age at NAKO baseline examination, sex, study center, and years of education. Age and sex were included as basic potential confounders. Previous studies have also demonstrated center-specific differences, e.g., in mental health complaints [3, 57, 58]. Thus, the study center was included as a design-based confounder. Finally, education was used as an indicator of the participants’ socio-economic position. Since sex and age were recently shown to moderate the associations between childhood maltreatment and the investigated diseases [7], stratified analyses for men and women and older and younger birth cohorts were tested. The birth year 1970 was used as the cut-off for the birth cohorts. It is close to the sample’s mean and median (1967) and represents a socio-politically relevant turning point in Germany.

## Results

The analyzed sample (Table 1) comprised 152,887 participants (77,297men, 50.56%) with a median age of 49 years (range: 19–75). Abuse was reported by 24,956 participants (16.32%), but more frequently among women than men (19.62% vs. 13.10%) and by the older compared to the younger cohort (17.76% vs. 14.03%). Neglect was reported by 22,826 participants (14.93%), with similar rates in men and women (14.31% vs. 15.56%), but much more frequently in the older than the younger cohort (18.58% vs. 9.12%). On average, 52.80% ever smoked, with a mean smoking initiation age of 17.90 years. Since smokers were identified before diagnosis, the proportion of smokers (range: 52.50%−52.78%) and the age at smoking initiation (range: 17.59–18.58) varied slightly between diseases. Of the participants who smoked before the respective diagnosis, 34.14% still smoked at the time of diagnosis (disease range: 30.85%−32.10%). While 17.09% reported early-onset overweight, 26.84% reported late-onset overweight, with slight variations between diseases (early-onset overweight range: 16.42%−16.87%; late-onset overweight range: 27.74%−28.96%). Excluding participants with early overweight only due to an increased weight compared to peers at age 10 reduced the frequency of early-onset overweight to 9.32% (disease range: 9.09%−9.31%).

**Table 1:** Descriptive statistics of the analyzed NAKO sample (*N* = 152,887), men (*n* = 77,297), women (*n* = 75,590), the older cohort (*n* = 93,963), and the younger cohort (*n* = 58,924)

	Whole Sample	Men	Women	Men vs. Women	Older cohort (born < = 1970)	Younger cohort (born > 1970)	Older vs. Younger
	*M [25%; 75%] %*	*M [25%; 75%] %*	*M [25%; 75%] %*	*d/V*	*M [25%; 75%]* *%*	*M [25%; 75%]* * %*	*d/V*
Age [Years]	49.16 [42.00; 59.00]	49.45 [42.00; 59.00]	48.87 [41.00; 59.00]	−0.05	57.12 [51.00; 63.00]	36.47 [30.00; 43.00]	−2.89
Sex [% Women]	49.44				49.27	49.72	4.35e-03
Education [Years]	15.70 [13.00; 18.00]	15.89 [13.00; 18.00]	15.52 [13.00; 17.00]	−0.16	15.62 [13.00; 17.00]	15.83 [13.00; 18.00]	0.09
Childhood abuse [% Yes]	16.32	13.10	19.62	0.09	17.76	14.03	0.05
Childhood neglect [% Yes]	14.93	14.31	15.56	0.02	18.58	9.12	0.13
Childhood maltreatment [% Yes]	25.82	23.19	28.52	0.06	29.66	19.70	0.11
Ever smoking [% Yes]	52.80	56.69	48.83	0.08	55.83	47.96	0.08
Current smoking [% Yes]	34.14	37.33	31.10	0.06	34.22	34.02	1.84e-03
Smoking initiation age [Years]	17.90 [15.00; 19.00]	17.89 [15.00; 19.00]	17.90 [15.00; 19.00]	1.27e-03	18.37 [15.00; 19.00]	17.02 [15.00; 18.00]	−0.24
Early overweight [% Yes]	17.09	19.01	15.13	0.05	16.22	18.49	0.03
Early overweight 18+ [% Yes]	9.32	11.55	7.02	0.08	8.08	11.33	0.05
Late overweight [% Yes]	26.84	34.30	19.22	0.17	35.16	13.58	0.24
Any cancer (*n* = 147,874) [% Yes]	3.88	3.12	4.67	0.04	5.88	0.78	0.13
Age at diagnosis [Years]	48.94 [41.00; 58.00]	52.42 [46.00; 61.00]	46.53 [39.00; 55.00]	−0.52	50.37 [44.00; 59.00]	31.97 [27.00; 37.00]	−1.73
Smoking-rel. cancer (*n* = 147,874) [% Yes]	1.81	0.80	2.85	0.08	2.75	0.34	0.09
Age at diagnosis [Years]	47.76 [41.00; 56.00]	53.70 [48.00; 60.00]	46.04 [39.00; 54.00]	−0.72	48.91 [43.00; 57.00]	33.28 [28.00; 39.00]	−1.52
Obesity-rel. cancer (*n* = 147,874) [% Yes]	2.85	2.31	3.41	0.03	4.40	0.44	0.11
Age at diagnosis [Years]	51.07 [45.00; 59.00]	54.19 [48.00; 61.00]	48.87 [43.00; 56.00]	−0.53	52.19 [46.00; 59.00]	33.57 [29.00; 40.00]	−1.98
MI (*n* = 152,666) [% Yes]	1.33	2.20	0.44	0.08	2.07	0.15	0.08
Age at diagnosis [Years]	51.79 [46.00; 58.00]	51.77 [46.00; 58.00]	51.91 [46.00; 59.00]	0.02	52.40 [47.00; 59.00]	38.23 [36.00; 42.00]	−1.64
Stroke (*n* = 152,607) [% Yes]	1.22	1.51	0.93	0.03	1.80	0.31	0.07
Age at diagnosis [Years]	51.21 [45.00; 59.00]	52.36 [46.00; 60.00]	49.29 [42.00; 59.00]	−0.29	52.94 [47.00; 60.00]	35.06 [30.00; 41.00]	−1.92
Diabetes (*n* = 150,935) [% Yes]	3.97	5.09	2.81	0.06	6.06	0.61	0.14
Age at diagnosis [Years]	51.93 [46.00; 59.00]	52.01 [46.00; 59.00]	51.79 [46.00; 59.00]	−0.02	52.86 [48.00; 59.00]	37.08 [34.00; 42.00]	−1.88
COPD (*n* = 149,469) [% Yes]	2.83	2.64	3.03	0.01	4.01	0.94	0.09
Age at diagnosis [Years]	43.07 [32.00; 54.00]	44.30 [33.00; 55.75]	41.97 [30.00; 53.00]	−0.17	45.04 [35.00; 55.50]	29.63 [22.00; 36.00]	−1.21
Anxiety (*n* = 151,761) [% Yes]	6.98	4.99	9.02	0.08	7.57	6.05	0.03
Age at diagnosis [Years]	38.81 [29.00; 48.00]	39.49 [30.00; 48.00]	38.42 [29.00; 47.00]	−0.09	43.10 [35.00; 51.00]	30.22 [24.00; 36.00]	−1.27
Depression (*n* = 151,089) [% Yes]	13.37	9.81	17.03	0.11	15.12	10.57	0.06
Age at diagnosis [Years]	39.95 [30.00; 49.00]	40.86 [31.00; 50.00]	39.41 [30.00; 48.00]	−0.12	44.09 [37.00; 52.00]	30.44 [24.00; 36.00]	−1.39

*MI* Myocardial infarction, *diabetes T*ype 2 diabete*s, COPD *Chronic bronchitis or chronic obstructive pulmonary disease, *Anxiety *Anxiety disorder or panic attacks, *M* Mean, *SD* Standard deviation *d Cohen’s d, V* Cramer’sV, Smoking and overweight were defined as disease-specific

### Childhood maltreatment and diseases

#### Childhood abuse

**Fig. 1:**
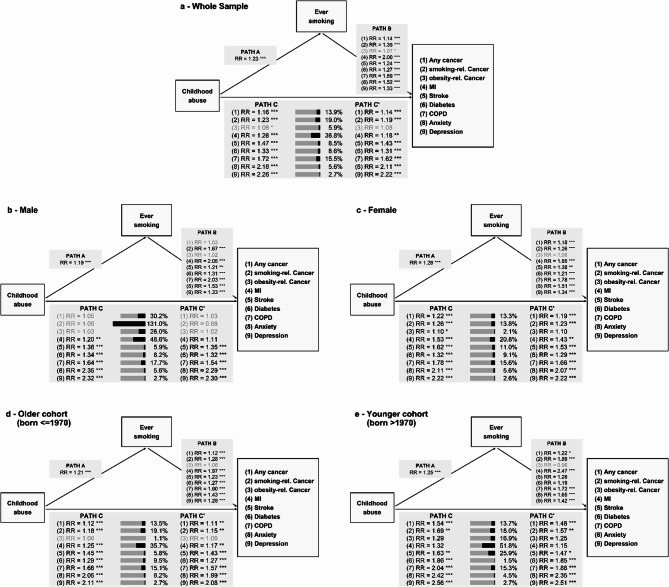
Mediation analyses for childhood abuse through smoking on the presence of the diseases. Mediation results are shown for the whole sample (panel a, top), men (panel b, middle left), women (panel c, middle right), the older cohort (panel d, bottom left) and the younger cohort (panel e, bottom right). Path C presents the total effect of childhood abuse on each disease. The stacked bars next to Path C present the direct and indirect effects with the black segment being the proportion mediated and the grey segment the complementary direct share. Path C' represents the pure direct effect of childhood abuse, independent of the smoking status. The four-way decomposition of the mediation model is presented in Table S8. Effects defined as non-substantive (RR<0.9 or RR>1.1) are printed in grey. Regression and mediation models were adjusted for age at NAKO baseline examination, study center, and educational years. Sex was used as a covariate for the whole sample and the birth cohort subgroups. Smoking status was defined before disease diagnosis. rel.=related; MI=myocardial infarction; diabetes=type 2 diabetes; COPD=chronic bronchitis or chronic obstructive pulmonary disease; Anxiety=anxiety disorder or panic attacks; *** p<0.001; ** p<0.01; * p<0.05

**Fig. 2:**
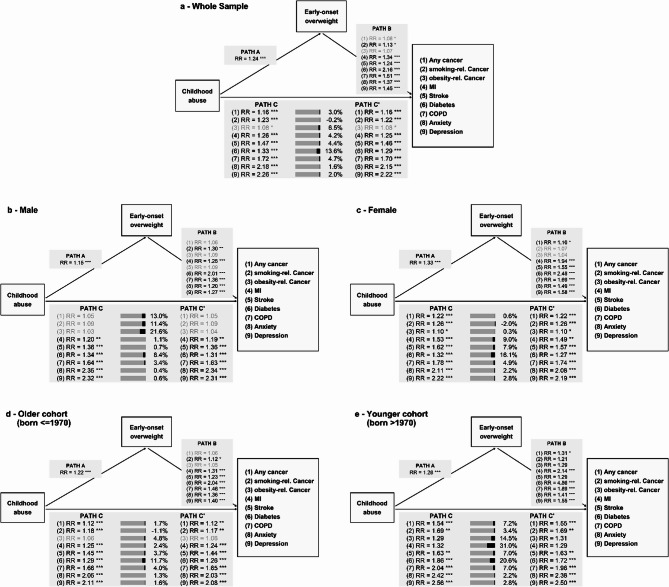
Mediation analyses for childhood abuse through early-onset overweight on the presence of the diseases. Mediation results are shown for the whole sample (panel a, top), men (panel b, middle left), women (panel c, middle right), the older cohort (panel d, bottom left) and the younger cohort (panel e, bottom right). Path C presents the total effect of childhood abuse on each disease. The stacked bars next to Path C present the direct and indirect effects with the black segment being the proportion mediated and the grey segment the complementary direct share. Path C' represents the pure direct effect of childhood abuse, independent of the overweight status. The four-way decomposition of the mediation model is presented in Table S10. Effects defined as non-substantive (RR<0.9 or RR>1.1) are printed in grey. Regression and mediation models were adjusted for age at NAKO baseline examination, study center, and educational years. Sex was used as a covariate for the whole sample and the birth cohort subgroups. Smoking status was defined before disease diagnosis. rel.=related; MI=myocardial infarction; diabetes=type 2 diabetes; COPD=chronic bronchitis or chronic obstructive pulmonary disease; Anxiety=anxiety disorder or panic attacks; *** p<0.001; ** p<0.01; * p<0.05

Participants who reported abuse, compared to those without, were more likely to report any diagnosis (RR range: 1.08–2.26; Figs. 1 and 2). Stronger associations were observed in the younger than the older birth cohort (Table 2). For cancer, substantive associations were found in women (RR range: 1.10–1.26) but not in men (RR range: 1.03–1.09). The mean age at depression diagnosis (*n* = 20,198) was 1.5 years earlier for abused participants (β=−1.54 [−1.81; −1.27]). Any cancer (*n* = 5,740), smoking-related cancer (*n* = 2,674), stroke (*n* = 1,864), and anxiety (*n* = 10,594) were diagnosed 6 to 9 months earlier. Effects for obesity-related cancer (*n* = 4,217), MI (*n* = 2,030), diabetes (*n* = 5,993), and COPD (*n* = 4,233) were even more negligible (Table 2).

**Table 2:** Total effects between childhood abuse and childhood neglect and the diseases

		Whole sample	Men	Women	Older cohort (born < = 1970)	Younger cohort (born > 1970)
		RR/β [95%-CI]	RR/β [95%-CI]	RR/β [95%-CI]	RR/β [95%-CI]	RR/β [95%-CI]
Childhood abuse	*The Presence of the disease*					
	Any cancer	1.16 [1.09; 1.24]	1.05 [0.94; 1.17]	1.22 [1.13; 1.31]	1.12 [1.05; 1.20]	1.54 [1.24; 1.92]
	Smoking-rel. cancer	1.23 [1.13; 1.35]	1.09 [0.88; 1.36]	1.26 [1.14; 1.39]	1.18 [1.08; 1.30]	1.69 [1.23; 2.31]
	Obesity-rel. cancer	1.08 [1.01; 1.17]	1.03 [0.91; 1.17]	1.10 [1.01; 1.21]	1.06 [0.98; 1.15]	1.29 [0.95; 1.75]
	MI	1.26 [1.13; 1.41]	1.20 [1.06; 1.36]	1.53 [1.21; 1.95]	1.25 [1.12; 1.40]	1.32 [0.77; 2.26]
	Stroke	1.47 [1.32; 1.64]	1.36 [1.18; 1.57]	1.62 [1.37; 1.90]	1.45 [1.29; 1.62]	1.63 [1.15; 2.31]
	Diabetes	1.33 [1.25; 1.41]	1.34 [1.24; 1.44]	1.32 [1.19; 1.45]	1.29 [1.21; 1.37]	1.86 [1.47; 2.35]
	COPD	1.72 [1.61; 1.84]	1.64 [1.48; 1.81]	1.78 [1.63; 1.94]	1.66 [1.55; 1.79]	2.04 [1.69; 2.45]
	Anxiety	2.18 [2.10; 2.27]	2.35 [2.19; 2.51]	2.11 [2.01; 2.21]	2.05 [1.96; 2.15]	2.42 [2.26; 2.59]
	Depression	2.26 [2.20; 2.32]	2.32 [2.22; 2.43]	2.22 [2.15; 2.29]	2.11 [2.05; 2.18]	2.56 [2.44; 2.69]
	*Age at diagnosis*					
	Any cancer	−0.63 [−1.19; −0.08]	0.08 [−0.77; 0.92]	−1.00 [−1.71; −0.28]	−0.66 [−1.25; −0.06]	−0.69 [−2.04; 0.66]
	Smoking-rel. cancer	−0.85 [−1.64; −0.06]	0.28 [−1.14; 1.70]	−1.08 [−2.00; −0.17]	−0.95 [−1.80; −0.11]	−0.10 [−1.93; 1.73]
	Obesity-rel. cancer	−0.28 [−0.86; 0.29]	0.02 [−0.84; 0.89]	−0.50 [−1.25; 0.24]	−0.28 [−0.88; 0.33]	−0.93 [−2.66; 0.80]
	MI	−0.46 [−1.31; 0.40]	−0.70 [−1.67; 0.28]	0.41 [−1.51; 2.33]	−0.58 [−1.47; 0.31]	1.99 [−0.60; 4.58]
	Stroke	−0.58 [−1.41; 0.24]	−0.10 [−1.09; 0.90]	−1.26 [−2.62; 0.10]	−0.67 [−1.55; 0.22]	0.91 [−1.37; 3.19]
	Diabetes	−0.29 [−0.74; 0.15]	0.12 [−0.44; 0.69]	−0.88 [−1.61; −0.15]	−0.30 [−0.77; 0.17]	−0.25 [−1.49; 0.98]
	COPD	−0.30 [−1.08; 0.48]	−0.05 [−1.27; 1.17]	−0.50 [−1.51; 0.51]	−0.52 [−1.39; 0.35]	1.61 [0.21; 3.00]
	Anxiety	−0.77 [−1.15; −0.39]	−1.02 [−1.68; −0.36]	−0.65 [−1.11; −0.18]	−0.94 [−1.47; −0.42]	−0.36 [−0.80; 0.07]
	Depression	−1.54 [−1.81; −1.27]	−1.50 [−1.96; −1.03]	−1.59 [−1.92; −1.26]	−1.87 [−2.22; −1.51]	−0.79 [−1.11; −0.47]
Childhood neglect	*The Presence of the disease*					
	Any cancer	1.08 [1.02; 1.15]	1.04 [0.95; 1.15]	1.10 [1.02; 1.20]	1.06 [1.00; 1.13]	1.42 [1.09; 1.84]
	Smoking-rel. cancer	1.12 [1.02; 1.22]	1.11 [0.91; 1.35]	1.11 [1.00; 1.23]	1.07 [0.98; 1.18]	1.89 [1.34; 2.67]
	Obesity-rel. cancer	1.01 [0.94; 1.09]	0.98 [0.88; 1.11]	1.02 [0.93; 1.13]	1.01 [0.93; 1.08]	1.10 [0.75; 1.61]
	MI	1.01 [0.91; 1.13]	0.93 [0.82; 1.04]	1.50 [1.17; 1.91]	1.00 [0.90; 1.12]	1.37 [0.79; 2.37]
	Stroke	1.35 [1.21; 1.49]	1.28 [1.12; 1.47]	1.45 [1.22; 1.71]	1.29 [1.16; 1.44]	2.18 [1.52; 3.12]
	Diabetes	1.09 [1.02; 1.15]	1.05 [0.98; 1.13]	1.15 [1.04; 1.27]	1.07 [1.01; 1.14]	1.46 [1.11; 1.91]
	COPD	1.40 [1.31; 1.50]	1.22 [1.09; 1.35]	1.57 [1.43; 1.72]	1.37 [1.27; 1.47]	1.73 [1.39; 2.14]
	Anxiety	1.73 [1.66; 1.80]	1.70 [1.58; 1.83]	1.74 [1.65; 1.84]	1.69 [1.61; 1.77]	1.83 [1.69; 2.00]
	Depression	1.75 [1.70; 1.81]	1.66 [1.58; 1.75]	1.81 [1.74; 1.87]	1.69 [1.64; 1.75]	1.96 [1.85; 2.08]
	*Age at diagnosis*					
	Any cancer	0.03 [−0.53; 0.59]	0.53 [−0.27; 1.34]	−0.29 [−1.06; 0.48]	0.06 [−0.53; 0.65]	−0.83 [−2.53; 0.88]
	Smoking-rel. cancer	−0.61 [−1.44; 0.21]	−0.65 [−2.08; 0.79]	−0.60 [−1.58; 0.39]	−0.61 [−1.48; 0.26]	−0.18 [−2.17; 1.81]
	Obesity-rel. cancer	0.26 [−0.32; 0.85]	0.26 [−0.59; 1.12]	0.32 [−0.48; 1.12]	0.28 [−0.32; 0.88]	−1.25 [−3.63; 1.13]
	MI	−0.15 [−1.00; 0.70]	−0.02 [−0.97; 0.94]	−0.45 [−2.22; 1.33]	−0.16 [−1.04; 0.71]	0.67 [−2.34; 3.69]
	Stroke	−0.42 [−1.20; 0.37]	0.04 [−0.87; 0.95]	−1.14 [−2.56; 0.28]	−0.52 [−1.36; 0.32]	−0.07 [−2.05; 1.90]
	Diabetes	−0.21 [−0.66; 0.24]	0.02 [−0.55; 0.60]	−0.56 [−1.27; 0.15]	−0.20 [−0.67; 0.27]	−0.16 [−1.38; 1.06]
	COPD	−0.30 [−1.12; 0.52]	−1.02 [−2.28; 0.25]	0.28 [−0.80; 1.36]	−0.44 [−1.34; 0.45]	1.18 [−0.49; 2.85]
	Anxiety	−0.39 [−0.82; 0.04]	−0.33 [−1.04; 0.38]	−0.42 [−0.96; 0.12]	−0.35 [−0.90; 0.19]	−0.54 [−1.10; 0.02]
	Depression	−0.83 [−1.13; −0.53]	−0.89 [−1.39; −0.39]	−0.80 [−1.17; −0.42]	−0.96 [−1.33; −0.59]	−0.48 [−0.88; −0.08]

*rel. *Related,* MI *Myocardial infarction,* diabetes *Type 2 diabetes,* COPD *Chronic bronchitis or chronic obstructive pulmonary disease,* Anxiety *Anxiety disorder or panic attacks,* RR *Risk ratio, *β *Unstandardized linear regression coefficient,* 95%-CI *95% confidence interval

#### Childhood neglect

Participants who reported neglect, compared to those without, were more likely to report any disease (RR range: 1.08–1.75; Table 2), with negligible effects for obesity-related cancer (RR = 1.01 [0.94; 1.09]) and MI (RR = 1.01 [0.91; 1.13]). However, a higher chance of MI was found in women (RR = 1.50 [1.17; 1.91]). Additionally, stronger associations were observed in the younger than the older birth cohort (Table 2). The mean age at depression diagnosis was 10 months earlier for neglected participants (β=−0.83 [−1.13; −0.53]). More negligible effects were found for the remaining diseases (Table 2).

Given the limited impact of abuse and neglect on the mean age at diagnosis (i.e., less than 1-year difference), further analyses focusing on this outcome were omitted.

### Smoking

Participants were more likely to smoke and to continue smoking till the diagnosis if they reported either abuse (ever smoker: RR = 1.23 [1.22; 1.24], current smoker: RR = 1.46 [1.44; 1.49]) or neglect (ever smoker: RR = 1.10 [1.09; 1.12], current smoker: RR = 1.27 [1.24; 1.29]). For abuse, the associations were similar for both sexes and cohorts. For neglect, slightly stronger associations were found for women (ever smoker: RR = 1.15 [1.13; 1.17], current smoker: RR = 1.38 [1.34; 1.42]) than men (ever smoker: RR = 1.06 [1.04; 1.07], current smoker: RR = 1.16 [1.13; 1.19]). Abused participants started smoking 7 months earlier than non-abused participants (β=−0.60 [−0.70; −0.49]), with similar effects for sexes and birth cohorts. Neglect did not influence the age at smoking initiation (β=−0.02 [−0.13; 0.10]).

Ever smokers, compared to never smokers, were more likely to report any disease (RR-range: 1.07-2.00; Fig. 1, Table S2). While ever smoking was linked to any cancer in women (RR = 1.18 [1.11; 1.26]), but negligible in men (RR = 1.03 [0.95; 1.12]), its association with smoking-related cancer was more pronounced in men (RR = 1.67 [1.39; 2.01]) than women (RR = 1.26 [1.16; 1.37]). Restricting the sample to current smokers before the diagnosis further increased the effect sizes for all diseases (RR-range: 1.44–4.48; Table S2). In contrast, younger smoking initiation, as an approximation of smoking intensity, showed weak disease associations (RR range: 0.96–0.99; Table S2), so mediation analyses for this mediator were omitted.

#### Mediation analyses with smoking

For abuse, small indirect effects through ever smoking were observed for all diseases (Fig. 1; Table S3). There was limited indication that exposure–mediator interaction substantially modified the effects (Table S8). The proportion mediated was higher for somatic diseases (5.88%−36.88%) than for mental disorders (2.66%−5.61%). For MI, one-third of the total effect was mediated through ever smoking (TIE: RR = 1.09 [1.06; 1.12], 36.88% [17.88%; 55.89%]), the highest in the sample. This mediation was sex-dependent: nearly 50% of the total effect was mediated in men (TIE: RR = 1.09 [1.06; 1.12], 48.62% [14.28%; 82.97%]) vs. 20% in women (TIE: RR = 1.08 [1.02; 1.14], 20.82% [2.75%; 38.89%]). For the whole sample, mediation by ever smoking was slightly stronger for smoking-related (RR = 1.04 [1.02; 1.06], 19.07% [6.97%; 31.16%]) compared to any cancer (RR = 1.02 [1.01; 1.03], 13.90% [2.33%; 25.47%]). Proportions mediated through current smoking were larger than for ever smoking, with similar increases over somatic diseases (range: 23.65%−62.71%) and mental disorders (range: 13.35%−18.39%). (Table S6). Again, there was limited indication of substantial exposure–mediator interactions (Table S9). For example, MI had the largest proportions mediated (TIE: RR = 1.25 [1.21; 1.29], 62.71% [43.61%; 81.80%]), with higher effects in men (TIE: RR = 1.25 [1.20; 1.30], 76.40% [42.00%; 110.80%]) than in women (TIE: RR = 1.23 [1.14; 1.20], 39.81% [21.68%; 57.94%]). Detailed results of the sensitivity analyses are presented in Additional file 1.

For neglect, the indirect effects through ever smoking were mostly negligible (Table 3). Analogous to abuse, however, the proportions mediated were higher for somatic diseases (3.49%−87.98%) than for mental disorders (2.24% and 3.83%). Note that some mediation estimates had extremely wide CIs due to the negligible total effects (RR ≈ 1.00). For substantive total effects, the highest proportions mediated were found for smoking-related cancer (TIE: RR = 1.01 [1.00; 1.02], 13.97% [−0.41%; 28.35%]) and COPD (TIE: RR = 1.03 [1.02; 1.04], 10.62% [7.40%; 13.84%]) in the whole sample. Similar to abuse, proportions mediated through current smoking were larger than for ever smoking (Additional file 1: Sensitivity analyses; Table S6).

**Table 3:** Mediation analyses between childhood neglect and the presence of diseases through smoking and early-onset overweight

		Whole sample			Men			Women			Older cohort (born < = 1970)			Younger cohort (born > 1970)		
		PDE	TIE		PDE	TIE		PDE	TIE		PDE	TIE		PDE	TIE	
		RR [95%-CI]	RR [95%-CI]	% [95%-CI]	RR [95%-CI]	RR [95%-CI]	% [95%-CI]	RR [95%-CI]	RR [95%-CI]	% [95%-CI]	RR [95%-CI]	RR [95%-CI]	% [95%-CI]	RR [95%-CI]	RR [95%-CI]	% [95%-CI]
Smoking	Any cancer	1.08 [1.01; 1.15]	1.01 [1.00; 1.01]	**8.15** **[−2.55; 18.85]**	1.04 [0.93; 1.15]	1.00 [1.00; 1.01]	**6.90** **[−20.73; 34.53]**	1.10 [1.01; 1.20]	1.01 [1.00; 1.02]	**8.10** **[−5.55; 21.74]**	1.06 [0.99; 1.13]	1.00 [1.00; 1.01]	**6.47** **[−5.89; 18.83]**	1.37 [1.04; 1.79]	1.04 [1.00; 1.07]	**11.74** **[−2.40; 25.88]**
	Smoking-rel. cancer	1.10 [1.00; 1.21]	1.01 [1.00; 1.02]	**13.97** **[−0.41; 28.35]**	1.14 [0.93; 1.39]	1.01 [1.00; 1.02]	**7.58** **[−8.15; 23.30]**	1.10 [0.98; 1.22]	1.02 [1.00; 1.03]	**16.63** **[−5.41; 38.67]**	1.06 [0.96; 1.17]	1.01 [1.00; 1.02]	**13.85** **[−9.05; 36.75]**	1.75 [1.23; 2.50]	1.07 [1.03; 1.12]	**14.29** **[3.93; 24.66]**
	Obesity-rel. cancer	1.01 [0.93; 1.09]	1.00 [0.99; 1.01]	**21.90** **[−127.56; 171.36]**	0.97 [0.86; 1.10]	1.01 [1.00; 1.01]	**−20.11** **[−115.05; 74.84]**	1.04 [0.94; 1.15]	1.00 [0.98; 1.01]	**−12.60** **[−68.40; 43.21]**	1.00 [0.93; 1.09]	1.00 [0.99; 1.01]	**18.83** **[−250.72; 288.38]**	1.07 [0.72; 1.58]	1.03 [0.97; 1.08]	**29.41** **[−104.08; 162.90]**
	MI	1.00 [0.90; 1.12]	1.04 [1.02; 1.05]	**87.98** **[−156.17; 332.12]**	0.92 [0.81; 1.05]	1.02 [1.01; 1.03]	**−37.98** **[−122.94; 46.99]**	1.42 [1.11; 1.82]	1.06 [1.02; 1.09]	**15.89** **[3.43; 28.35]**	0.99 [0.89; 1.11]	1.03 [1.02; 1.04]	**130.45** **[−494.17; 755.08]**	1.30 [0.73; 2.32]	1.06 [0.98; 1.14]	**20.42** **[−21.53; 62.37]**
	Stroke	1.34 [1.20; 1.50]	1.01 [1.00; 1.02]	**3.49** **[−0.94; 7.92]**	1.27 [1.10; 1.46]	1.01 [1.00; 1.02]	**3.70** **[−1.33; 8.72]**	1.46 [1.23; 1.74]	1.01 [0.98; 1.03]	**1.88** **[−5.39; 9.15]**	1.29 [1.15; 1.44]	1.01 [1.00; 1.02]	**2.84** **[−1.89; 7.56]**	2.09 [1.46; 2.99]	1.03 [0.99; 1.08]	**6.14** **[−2.59; 14.87]**
	Diabetes	1.09 [1.02; 1.16]	1.01 [1.00; 1.01]	**8.51** **[−0.86; 17.88]**	1.07 [0.99; 1.16]	1.00 [1.00; 1.01]	**4.36** **[−5.18; 13.91]**	1.13 [1.02; 1.25]	1.02 [1.00; 1.03]	**12.98** **[−1.71; 27.67]**	1.07 [1.01; 1.14]	1.01 [1.00; 1.01]	**9.00** **[−2.22; 20.22]**	1.46 [1.10; 1.93]	1.01 [0.97; 1.04]	**1.66** **[−10.40; 13.73]**
	COPD	1.37 [1.27; 1.47]	1.03 [1.02; 1.04]	**10.62** **[7.40; 13.84]**	1.20 [1.08; 1.34]	1.02 [1.01; 1.03]	**11.86** **[4.09; 19.64]**	1.52 [1.38; 1.67]	1.04 [1.03; 1.06]	**11.21** **[7.01; 15.41]**	1.34 [1.24; 1.44]	1.03 [1.02; 1.04]	**9.82** **[6.44; 13.21]**	1.63 [1.31; 2.04]	1.05 [1.02; 1.08]	**11.27** **[3.48; 19.06]**
	Anxiety	1.70 [1.63; 1.78]	1.02 [1.01; 1.02]	**3.83** **[2.67; 4.99]**	1.68 [1.55; 1.81]	1.01 [1.01; 1.02]	**3.56** **[1.92; 5.21]**	1.74 [1.64; 1.85]	1.02 [1.01; 1.03]	**4.14** **[2.33; 5.94]**	1.66 [1.58; 1.75]	1.01 [1.01; 1.02]	**3.51** **[2.26; 4.77]**	1.80 [1.65; 1.97]	1.02 [1.01; 1.03]	**4.23** **[1.56; 6.91]**
	Depression	1.74 [1.68; 1.80]	1.01 [1.01; 1.01]	**2.24** **[1.47; 3.02]**	1.65 [1.56; 1.75]	1.01 [1.00; 1.01]	**1.97** **[0.90; 3.03]**	1.81 [1.73; 1.88]	1.01 [1.01; 1.02]	**2.62** **[1.43; 3.81]**	1.68 [1.61; 1.74]	1.01 [1.01; 1.01]	**2.23** **[1.37; 3.09]**	1.95 [1.82; 2.08]	1.01 [1.00; 1.02]	**2.01** **[0.33; 3.69]**
Early-onset Overweight	Any cancer	1.08 [1.01; 1.15]	1.00 [1.00; 1.00]	**1.18** **[−1.86; 4.22]**	1.10 [0.91; 1.34]	1.00 [1.00; 1.00]	**0.51** **[−1.84; 2.87]**	1.10 [1.01; 1.20]	1.00 [1.00; 1.00]	**−0.27** **[−4.81; 4.28]**	1.06 [0.99; 1.14]	1.00 [1.00; 1.00]	**1.13** **[−2.60; 4.86]**	1.40 [1.08; 1.83]	1.00 [0.99; 1.01]	**0.83** **[−3.06; 4.73]**
	Smoking-rel. cancer	1.11 [1.01; 1.22]	1.00 [1.00; 1.00]	**0.78** **[−2.49; 4.06]**	0.99 [0.88; 1.11]	1.00 [1.00; 1.00]	**−5.81** **[−70.52; 58.89]**	1.11 [1.00; 1.24]	1.00 [0.99; 1.01]	**0.37** **[−5.05; 5.78]**	1.07 [0.97; 1.18]	1.00 [1.00; 1.00]	**0.78** **[−4.06; 5.62]**	1.87 [1.31; 2.66]	1.00 [0.99; 1.02]	**0.26** **[−2.85; 3.36]**
	Obesity-rel. cancer	1.01 [0.94; 1.09]	1.00 [1.00; 1.00]	**8.46** **[−45.90; 62.82]**	0.92 [0.81; 1.04]	1.00 [1.00; 1.00]	**−0.17** **[−1.58; 1.23]**	1.02 [0.93; 1.13]	1.00 [0.99; 1.00]	**−2.08** **[−27.38; 23.23]**	1.01 [0.93; 1.09]	1.00 [1.00; 1.00]	**12.29** **[−139.86; 164.45]**	1.10 [0.74; 1.61]	1.01 [0.99; 1.02]	**5.46** **[−20.76; 31.69]**
	MI	1.01 [0.90; 1.12]	1.00 [1.00; 1.01]	**25.09** **[−261.47; 311.66]**	1.28 [1.11; 1.46]	1.00 [1.00; 1.00]	**0.01** **[−0.52; 0.53]**	1.48 [1.16; 1.88]	1.01 [1.00; 1.03]	**4.25** **[−0.66; 9.17]**	1.00 [0.89; 1.12]	1.00 [1.00; 1.01]	**271.94**	1.34 [0.76; 2.35]	1.01 [0.98; 1.03]	**2.46** **[−7.71; 12.64]**
	Stroke	1.35 [1.21; 1.50]	1.00 [1.00; 1.01]	**1.87** **[0.12; 3.63]**	1.05 [0.97; 1.14]	1.00 [1.00; 1.01]	**4.94** **[−9.86; 19.74]**	1.43 [1.20; 1.69]	1.02 [1.01; 1.03]	**6.16** **[1.60; 10.73]**	1.29 [1.16; 1.44]	1.00 [1.00; 1.01]	**1.89** **[−0.16; 3.94]**	2.18 [1.53; 3.12]	1.01 [0.99; 1.02]	**1.48** **[−1.50; 4.46]**
	Diabetes	1.08 [1.01; 1.15]	1.01 [1.01; 1.02]	**14.96** **[3.36; 26.56]**	1.22 [1.09; 1.36]	1.00 [1.00; 1.00]	**0.74** **[−1.21; 2.70]**	1.13 [1.02; 1.25]	1.02 [1.01; 1.03]	**17.01** **[3.84; 30.17]**	1.06 [1.00; 1.13]	1.01 [1.00; 1.02]	**15.47** **[0.60; 30.34]**	1.44 [1.09; 1.89]	1.04 [1.01; 1.08]	**11.97** **[1.02; 22.93]**
	COPD	1.40 [1.30; 1.50]	1.01 [1.00; 1.01]	**2.49** **[1.07; 3.92]**	1.70 [1.57; 1.83]	1.00 [1.00; 1.00]	**0.21** **[−0.27; 0.68]**	1.55 [1.41; 1.70]	1.01 [1.01; 1.02]	**3.61** **[1.60; 5.61]**	1.36 [1.26; 1.47]	1.01 [1.00; 1.01]	**2.32** **[0.77; 3.88]**	1.71 [1.37; 2.12]	1.01 [1.00; 1.03]	**3.12** **[−0.15; 6.39]**
	Anxiety	1.72 [1.64; 1.80]	1.01 [1.00; 1.01]	**1.32** **[0.66; 1.97]**	1.66 [1.57; 1.75]	1.00 [1.00; 1.00]	**0.29** **[−0.22; 0.79]**	1.73 [1.63; 1.83]	1.01 [1.01; 1.02]	**2.38** **[1.27; 3.48]**	1.68 [1.59; 1.77]	1.01 [1.00; 1.01]	**1.23** **[0.49; 1.97]**	1.82 [1.66; 1.99]	1.01 [1.00; 1.01]	**1.51** **[0.16; 2.85]**
	Depression	1.74 [1.68; 1.79]	1.01 [1.00; 1.01]	**1.52** **[0.91; 2.13]**	1.10 [0.91; 1.34]	1.00 [1.00; 1.00]	**0.51** **[−1.84; 2.87]**	1.79 [1.71; 1.86]	1.01 [1.01; 1.02]	**2.63** **[1.67; 3.59]**	1.68 [1.62; 1.74]	1.01 [1.00; 1.01]	**1.38** **[0.71; 2.05]**	1.94 [1.81; 2.07]	1.01 [1.00; 1.01]	**1.77** **[0.52; 3.01]**

Regression and mediation models were adjusted for age at NAKO baseline examination, study center, and educational years. Sex was used as a covariate for the whole sample and the birth cohort subgroups. Smoking and overweight status were defined before disease diagnosis. The four-way decomposition of the mediation models are presented in Table S8 and Table S10

*PDE* Pure direct effect, *TIE *Total indirect effect, *rel*. Related, *MI* Myocardial infarction, *diabetes* Type 2 diabetes, *COPD* Chronic bronchitis or chronic obstructive pulmonary disease, *Anxiety* Anxiety disorder or panic attacks, *RR* Risk ratio, *95%-CI* 95% confidence interval, *% *Proportion mediated

### Overweight

Abuse (RR = 1.24 [1.21; 1.28]) and neglect (RR = 1.10 [1.07; 1.13]) were associated with a higher chance of early-onset overweight, more pronounced in women (abuse: RR = 1.33 [1.28; 1.38]; neglect: RR = 1.19 [1.14; 1.24]) than in men (abuse: RR = 1.15 [1.11; 1.20]; neglect: RR = 1.03 [0.99; 1.07]). The effects were comparable between the birth cohorts. Excluding participants who were only overweight due to a higher weight compared to peers at age 10, effect sizes slightly lowered for both abuse (whole sample: RR = 1.22 [1.17; 1.27]) and neglect (whole sample: RR = 1.06 [1.01; 1.11]). Late-onset overweight (> 30 years) was not substantively associated with abuse (RR = 1.05 [1.03; 1.08]) or neglect (RR = 0.99 [0.97; 1.01]), so further analyses were omitted.

Early-onset overweight was associated with a higher chance of any disease (RR range: 1.07–2.16; Fig. 2, Table S4). While early-onset overweight was more strongly associated with smoking-related cancer in men (RR = 1.30 [1.08; 1.58]) than in women (RR = 1.07 [0.95; 1.20]), associations with other diseases were stronger in women (Fig. 2, Table S4). Additionally, effect sizes for MI and diabetes were larger in the younger (MI: RR = 2.14 [1.37; 3.33]; diabetes: RR = 4.86 [3.93; 6.01]) than the older birth cohort (MI: RR = 1.31 [1.18; 1.46]; diabetes: RR = 2.04 [1.93; 2.15]).

Excluding participants who were only overweight due to a higher weight compared to peers at age 10, effect sizes remained mostly stable (RR range: 1.03–2.35, Table S4). Nevertheless, mediation analyses were conducted as sensitivity analyses with this subgroup of adult early-onset overweight participants and are presented in Additional file 1.

#### Mediation analyses with overweight

For abuse, the proportions mediated by early-onset overweight were mostly negligible (−0.25%−6.51%; Fig. 2, Table S5), except for diabetes (TIE: RR = 1.04 [1.03; 1.04], 13.69% [9.85%; 17.52%]). With 16–20%, the proportion of diabetes mediated through early-onset overweight was roughly doubled in women (TIE: RR = 1.04 [1.03; 1.06], 16.16% [8.92%; 23.39%]) and in the younger birth cohort (RR = 1.11 [1.07; 1.15], 20.63% [12.67%; 28.59%]) compared to men (RR = 1.02 [1.01; 1.03], 8.43% [4.52%; 12.35%]) and the older birth cohort (RR = 1.03 [1.02; 1.04], 11.76% [7.58%; 15.95%]). There was limited indication of substantial exposure–mediator interactions (Table S10). Moreover, excluding participants who were only overweight due to a higher weight compared to peers at age 10, the proportions mediated further decreased (Table S7, Table S11). Detailed results of the sensitivity analyses are presented in Additional file 1.

Analogous to abuse, mediation effects for neglect were negligible (0.78%−8.46%; Table 3), except for diabetes (TIE: RR = 1.01 [1.01; 1.02], 14.96% [3.36%; 26.56%]), again with limited indication of substantial exposure-mediator interactions (Table S10). In women, 19% of the association between neglect and diabetes was explained by early-onset overweight (TIE: RR = 1.02 [1.01; 1.03], 17.01% [3.84%; 30.17%]). However, only 12% were mediated in the younger birth cohort (TIE: RR = 1.04 [1.01; 1.08], 11.97% [1.02%; 22.93%]). Note that the CIs for the proportion mediated by MI in the whole sample and the older cohort were extremely wide, including implausible values, most probably due to the negligible total effects. Excluding participants who were only overweight due to a higher weight compared to peers at age 10, the proportions mediated by diabetes decreased (TIE: RR = 1.00 [1.00; 1.01], 5.79% [−3.20%; 14.77%]). Other sensitivity mediation results were more comparable (Additional file 1: Sensitivity analyses; Table S7, Table S11).

## Discussion

Data from a large population-based study were used to investigate whether the associations between childhood maltreatment and seven diseases were mediated by smoking or early-onset overweight. Separate models were calculated for abuse and neglect. In contrast to earlier studies, we implemented a clear time sequence between mediators and outcomes to distinguish the effects of childhood maltreatment from the modifiable factors, i.e., smoking and overweight. Consistent with previous findings, we found stronger associations between abuse and the mediators and diseases than neglect [13–16]. Despite these differences in direct associations, the mediation effects of smoking and early-onset overweight were comparable for abuse and neglect. However, direct effects on disease outcomes remained, underscoring the need to explore additional pathways in future research.

Lang et al. [19] described smoking and obesity as “hallmarks” of childhood maltreatment. Accordingly, we found substantive indirect effects through ever smoking for somatic diseases, particularly MI, cancer, and COPD, which aligns with previous findings [21, 22, 59]. Moreover, the age at last smoking cessation was used to define current smokers at the time of the diagnosis. These sensitivity analyses yielded larger mediation effects, yet a residual direct effect of childhood maltreatment on the outcomes remained. Moreover, the definition of “current smoking” still included some uncertainty, as we could not rule out that the participant was within a smoking break at the time of diagnosis. In contrast, early-onset overweight showed a reliable, substantive indirect effect only for type 2 diabetes. Sensitivity analyses excluding participants with overweight solely at age 10 revealed an attenuation of this effect.

While we ensured that mediators preceded diagnosis in the present study, the temporal sequence between childhood maltreatment and mediators was less concise. As a result, overweight at age 10 and early smoking initiation may still coincide with maltreatment occurring in late childhood or adolescence. Besides, the age at smoking initiation clustered around the late adolescence and early adulthood and thus, according to a recent general-population study, is unlikely to be before childhood maltreatment onset [60]. In detail, participants started smoking at a mean age of 18 years, but fewer participants reported early-onset overweight (17%) than late-onset overweight (> 30 years; 27%). Overweight at age 10 was assessed compared to peers using broad categories (lower/regular/higher), which might have introduced more misclassification than the BMI-based classification used at later ages. Excluding participants who were classified as overweight solely based on the weight report at age 10 reduced the mediation effects, underscoring the importance of overweight in childhood and adolescence. Moreover, we found no association between abuse or neglect and later-onset overweight, suggesting that the greater temporal distance from childhood maltreatment weakens this link. In contrast, since many of the analyzed somatic diseases manifest later in life, early-onset overweight may not have been a strong enough mediator to explain the associations.

The interplay between smoking and weight-related coping mechanisms further complicates their roles as mediators, a complexity we accounted for by including exposure–mediator interaction terms in the mediation models. Smoking has been linked to lower weight [61], while both smoking and unhealthy diets, leading to obesity, have been discussed as coping mechanisms for emotional distress [27–29]. Additionally, in women with eating disorders, childhood maltreatment has been associated with a heightened drive for thinness and bulimia [62], highlighting individual variations in coping responses. These findings suggest that the pathways between childhood maltreatment, smoking, and overweight may interact with each other as well as personal factors. However, in the presented analyses, only negligible portions of the mediation effects were attributable to exposure–mediator interaction.

Moreover, we found sex-dependent mediation effects, especially for MI. In men, ever smoking explained 49% of abuse on MI, compared to 21% in women. These effects increased to 76% in men and 40% in women using current smoking as the mediator. Contrarily, 16% of the abuse effect on type 2 diabetes was mediated by early-onset overweight in women but only 8% in men. Similar sex differences have been shown for obesity and BMI in previous studies [5, 28, 63]. Sex-dependent biological and psychological responses to chronic stress, such as childhood maltreatment, might partly explain these differences [64, 65]. While male college students reported problem-oriented coping strategies, female fellows used emotion-oriented strategies more often [64, 66]. Nevertheless, avoidant coping strategies were associated with more severe symptoms in patients with heart failure [67].

Besides the female sex, older age, and lower educational attainment were identified as risk factors for multi-morbidity [68, 69]. Indeed, childhood maltreatment has been associated with a higher number of comorbidities in patients with mental disorders [4, 8, 70]. However, the exact connections remain unclear. A large longitudinal study reported that the association between childhood maltreatment and CVD was independent of depressive symptoms, while a review summarized that statistical adjustment for mental disorders attenuated the association between childhood maltreatment and CVD [10, 20]. Moreover, findings from cross-sectional studies often differed from those of longitudinal studies, providing an apparent time sequence between mediator and outcome [5, 20, 23–26]. In our study, we ensured that mediators preceded diagnoses by considering smoking initiation age, weight history, and age at diagnosis, thus implementing a longitudinal approach to the cross-sectional data. Additionally, we excluded participants with childhood diagnoses to avoid confounding with adverse childhood experiences [6].

Given the complex nature of the diseases, genetic predispositions likely play a significant role in developing those [71, 72]. Nevertheless, previous studies reported a younger age of onset and a more chronic, treatment-resistant disease course in maltreated patients for various mental disorders [8, 9]. Although it seems plausible that childhood maltreatment influences the timing of disease onset more strongly than its occurrence, the effects of childhood abuse and neglect on the age at diagnosis were minor in our results. In detail, depression began 1.5 years earlier if the participants reported abuse and one year earlier if they reported neglect. However, Nelson et al. [9] reported an average onset four years earlier in a meta-analysis. Our sample was drawn from the general population, which might explain smaller effect sizes, and used self-reported diagnoses, which might be delayed to the first symptoms or episodes [9, 57, 58].

Nevertheless, for all analyzed diseases, parts of the association with abuse and neglect remained unexplained by smoking and early-onset overweight. Hence, our results support that additional pathways are likely and may converge into a multi-mediator model, as Salzmann et al. (2022) already postulated for emotional childhood neglect. However, more in-depth phenotyping of the mediators and outcomes is necessary.

Among the limitations, the study had a relatively low response rate in NAKO and many missing values for the CTS [3, 37]; missing values were not imputed. Selection bias is also possible, as individuals with severe disease courses are less likely to participate in population-based studies. Moreover, the analyzed sample was younger and more highly educated than both the excluded participants and the German adult population [51]. Because higher educational attainment and higher socio-economic status are each linked to a lower prevalence of childhood maltreatment and better health outcomes [73, 74], the observed associations may underestimate the true effects in less educated or older participants; generalization to these groups should therefore be made with caution.

Additionally, relying on self-reported exposure, mediator, and outcome variables introduces the risk of recall bias. Childhood maltreatment was only assessed by five items, limiting precision. Moreover, detailed timing information on childhood maltreatment would be needed to refine the temporal sequence between exposure and mediator, but it was unavailable in NAKO. Age at diagnosis rather than symptom onset was used, potentially delaying disease classification, particularly for mental disorders [57]. Additionally, the age at diagnosis might depend on other factors not accounted for in this study, e.g., the frequency of doctor visits.

Although associations between age at smoking initiation and age at diagnosis were negligible in our sample, we cannot rule out that the intensity of smoking and overweight may also have influenced the mediation effects [59, 75]. For smoking, cumulative dose parameters such as pack-years were only available as lifetime markers and could not be positioned between childhood maltreatment and the diagnosis. Nevertheless, future research should include time-sensitive intensity markers to extend our results. Furthermore, weight history was only available at four ages, with large intervals, and BMI was used as a proxy for overweight, which does not account for variations in muscle mass [76]. Future studies should thus integrate intensity markers for both mediators and use more precise body fat assessments.

Finally, all analyses were adjusted for age at baseline, sex, study center, and education. Unfortunately, parental socio-economic status (SES) was not included in the analyzed dataset. Therefore, the impact of parental SES instead of participants’ education on the mediation models should be investigated in future studies. Age at baseline accounts for the increasing disease risk with age and captures birth cohort effects. Cohort effects influence exposure, mediators, and outcomes. We considered adjusting for years at risk, defined as age at baseline for unaffected participants and age at diagnosis for affected participants, effectively shifting the reference point for affected individuals forward. However, years at risk may be influenced by childhood maltreatment or the mediators. Due to high collinearity, adjusting simultaneously for age at baseline and years at risk is impossible. Future studies should explore alternative approaches to account for the influence of time on these associations.

## Conclusions

In summary, we used timing information in cross-sectional data to impose a clear time sequence between mediators and outcomes. This approach enabled us to quantify how much of maltreatment-related adult disease risk is attributable to modifiable factors, here smoking and overweight. It thus highlighted actionable targets for secondary prevention and intervention, while also underscoring the continued need for primary prevention of childhood maltreatment. Further, analyzing abuse and neglect separately revealed both common and inherent pathway differences.

Associations between childhood abuse and somatic or mental diseases were stronger than those with childhood neglect, but mediation via smoking and overweight was similar for both exposures. Smoking had substantive indirect effects on MI, type 2 diabetes, and COPD, while overweight-mediated effects were limited to type 2 diabetes. Missing evidence for overweight as a mediator may reflect the late age at diagnosis and the weak association between childhood maltreatment and later-onset overweight in our sample. Sex differences emerged, with stronger smoking-related mediation for MI in men and overweight-related mediation for type 2 diabetes in women.

Nevertheless, much of the childhood maltreatment–disease link remained unexplained, indicating additional pathways. Future research should refine mediation models with more precise measures of smoking intensity, body composition, and exposure timing. A multi-mediator approach may provide deeper insights into the complex pathways linking childhood maltreatment to disease risk.

## Supplementary Information

Supplementary Material 1. Sensitivity analyses and additional tables are presented in Additional file 1 (Additional_20250817.pdf). The file contains the mediation analyses with current smoking and early-onset overweight during adulthood (18+), respectively, as well as Tables S1-S11.

## Data Availability

The data underlying this study are not publicly available due to contractual restrictions. Researchers interested in accessing these data may apply through NAKO at (transfer.nako.de/transfer/index). The present project was approved by the Use and Access Committee of the General Assembly of NAKO (application number 776).
