# Supplementary material for: Childhood maltreatment and adult diseases in the general population: the mediating role of smoking and overweight in a time-sequence design
Source: BMC Public Health. 2025 Oct 27;25:3613. doi: 10.1186/s12889-025-24854-y (PMC12560492; doi:10.1186/s12889-025-24854-y)
Supplement: Supplementary file 1 — Supplementary Material 1. Sensitivity analyses and additional tables are presented in Additional file 1 (Additional_20250817.pdf). The file contains the mediation analyses with current smoking and early-onset overweight during adulthood (18+), respectively, as well as Tables S1-S11. [file 12889_2025_24854_MOESM1_ESM.docx]

Supplementary material

Childhood maltreatment and diseases in adulthood: the mediating role of smoking and overweight

Johanna Klinger-König*, Fabian Streit, Maja P. Völker, Josef Frank, Peggy Sekula, Stefanie Jaskulski, Michael Leitzmann, Claudia Meinke-Franze, Carsten O. Schmidt, Thomas Keil, Stefan N Willich, Tobias Pischon, Ilais M. Velásquez, Jonas Frost, Börge Schmidt, Jana-Kristin Heise, Carolina J. Klett-Tammen, Lena Koch-Gallenkamp, Nadia Obi, Volker Harth, Ute Mons, Klaus Berger, Karin H. Greiser, Rafael Mikolajczyk, Matthias B Schulze, Hans J Grabe

Sensitivity analyses

*Mediation analyses with current smoking*

Participants who reported both starting and quitting smoking before the diagnosis were excluded to conduct sensitivity analyses comparing never to current smokers.

For abuse, indirect effects through current smoking were larger than for ever smoking for all diseases (Table S6). The proportion mediated was higher for somatic diseases (23.65%-62.71%) than for mental disorders (13.35%-18.39%). There was limited indication that exposure–mediator interaction substantially modified the effects (Table S9). Comparable to ever smoking, the highest proportion mediated was found for MI (TIE: RR=1.25 [1.21; 1.29], 62.71% [43.61%; 81.80%]), with higher effects in men (TIE: RR=1.25 [1.20; 1.30], 76.40% [42.00%; 110.80%]) than in women (TIE: RR=1.23 [1.14; 1.32], 39.81% [21.68%; 57.94%]). For any cancer and smoking-related cancer, higher proportions mediated were observed for the older (ANY: TIE: RR=1.07 [1.05; 1.09], 46.32% [18.75%; 73.89%]; SMOKING-RELATED: TIE: RR=1.09 [1.06; 1.13], 52.31% [17.93%; 86.70%) compared to the younger cohort (ANY: TIE: RR=1.13 [1.06; 1.20], 29.37% [11.70%; 47.05%]; SMOKING-RELATED: TIE: RR=1.17 [1.08; 1.27], 32.51% [13.38%; 51.64%).

For neglect, indirect effects through current smoking were also larger than for ever smoking for all diseases (Table S6). Again, the proportions mediated were higher for somatic diseases (21.76%-49.92%) than for mental disorders (12.56%-16.44%) with limited indication of substantial exposure–mediator interactions (Table S9). Note that some mediation estimates, particularly for MI and obesity-related cancer, had extremely wide CIs due to the negligible total effects (RR≈1.00). For substantive total effects, the highest proportions mediated were found for smoking-related cancer (TIE: RR=1.07 [1.05; 1.09], 49.92% [12.23%; 87.60%]) with larger effects for women (TIE: RR=1.07 [1.04; 1.10], 60.19% [-0.90%; 121.28%]) than for men (TIE: RR=1.06 [1.03; 1.09], 24.03% [0.49%; 47.57%]) and the older (TIE: RR=1.05 [1.03; 1.08], 56.64% [-10.11%; 123.39%]) compared to the younger cohort (TIE: RR=1.15 [1.08; 1.23], 27.71% [10.86%; 44.57%]).

*Mediation analyses with early-onset overweight during adulthood (18+)*

Participants who were overweight before the diagnosis solely due to an increased weight compared to peers at age 10 were excluded to conduct sensitivity analyses.

For abuse, the proportions mediated in the subsample of early-onset overweight participants during adulthood were smaller compared to the complete sample (Table S7) with limited indication of substantial exposure–mediator interactions (Table S11). The proportions mediated were higher for somatic diseases (0.83%-9.91%) than for mental disorders (0.68%-1.36%). Highest proportions mediated were observed for diabetes (TIE: RR=1.03 [1.02; 1.03], 9.91% [6.48%; 13.35%]) with larger effects for women (TIE: RR=1.04 [1.02; 1.05], 14.20% [7.54%; 20.86%]) than men (TIE: RR=1.01 [1.00; 1.02], 3.94% [0.89%; 6.99%]) and for the younger (TIE: RR=1.10 [1.06; 1.14], 19.50% [10.91%; 28.08%]) compared to the older cohort (TIE: RR=1.02 [1.01; 1.03], 7.67% [4.02%; 11.31%]).

Similarly, the proportions mediated for neglect were smaller in the subsample (Table S7). However, the effects were still higher for somatic diseases (0.23%-5.79%) than for mental disorders (0.34% and 0.59%). There was limited indication that exposure–mediator interaction substantially modified the effects (Table S11). Again, some mediation estimates had extremely wide CIs due to the negligible total effects (RR≈1.00), particularly for MI and obesity-related cancer in the whole sample, men, and the older cohort. For substantive total effects, the highest proportions mediated were found for diabetes, but with negligible indirect effects (TIE: RR=1.00 [1.00; 1.01], 5.79% [-3.20%; 14.77%]).

Tables

**Table S1.** Descriptive statistics of the analyzed NAKO sample compared to the excluded sample.

|  | **Analytic sample** | | **Excluded sample** | |  |
| --- | --- | --- | --- | --- | --- |
|  | *N* | *Mean [25%; 75%] / %* | *N* | *Mean [25%; 75%] / %* | *d / V* |
| **Age** [Years] | 152887 | 49.16 [42.00; 59.00] | 51905 | 51.89 [44.00; 63.00] | 0.21 |
| **Sex** [% Women] | 152887 | 49.44 | 51905 | 53.45 | -0.03 |
| **Education** [Years] | 152887 | 15.70 [13.00; 18.00] | 33186 | 14.49 [13.00; 16.00] | -0.52 |
| **Childhood abuse** [% Yes] | 152887 | 16.32 | 19123 | 18.82 | -0.02 |
| **Childhood neglect** [% Yes] | 152887 | 14.93 | 19123 | 17.37 | -0.02 |
| **Childhood maltreatment** [% Yes] | 152887 | 25.82 | 19251 | 29.55 | -0.02 |
| **Ever smoking** [% Yes] | 152887 | 52.80 | 43009 | 57.28 | -0.04 |
| **Current smoking** [% Yes] | 109934 | 34.14 | 29157 | 36.99 | -0.02 |
| **Smoking initiation age** [Years] | 80725 | 17.90 [15.00; 19.00] | 23861 | 18.51 [15.00; 19.00] | -2.13e-03 |
| **Early overweight** [% Yes] | 152887 | 17.09 | 18708 | 22.70 | -0.04 |
| **Early overweight 18+** [% Yes] | 152887 | 9.32 | 18708 | 14.33 | -0.04 |
| **Late overweight** [% Yes] | 152887 | 26.84 | 14257 | 50.00 | -0.13 |
| **Any cancer** [% Yes] | 147885 | 3.88 | 49562 | 5.06 | -0.02 |
| **Age at diagnosis** [Years] | 5751 | 48.94 [41.00; 58.00] | 2507 | 50.69 [43.00; 60.00] | -0.01 |
| **Smoking-rel. cancer** [% Yes] | 147885 | 1.81 | 49562 | 2.58 | -0.02 |
| **Age at diagnosis** [Years] | 2677 | 47.76 [41.00; 56.00] | 1279 | 49.83 [42.00; 59.00] | 8.39e-04 |
| **Obesity-rel. cancer** [% Yes] | 147885 | 2.85 | 49562 | 3.75 | -0.02 |
| **Age at diagnosis** [Years] | 4223 | 51.07 [45.00; 59.00] | 1858 | 52.51 [46.00; 60.00] | -5.09e-05 |
| **MI** [% Yes] | 152675 | 1.33 | 51473 | 2.54 | -0.04 |
| **Age at diagnosis** [Years] | 2039 | 51.79 [46.00; 58.00] | 1309 | 51.94 [46.00; 59.00] | -7.04e-04 |
| **Stroke** [% Yes] | 152608 | 1.22 | 51348 | 2.26 | -0.04 |
| **Age at diagnosis** [Years] | 1865 | 51.21 [45.00; 59.00] | 1163 | 52.29 [46.00; 60.00] | -0.01 |
| **Diabetes** [% Yes] | 150950 | 3.97 | 50886 | 7.72 | -0.07 |
| **Age at diagnosis** [Years] | 6008 | 51.93 [46.00; 59.00] | 3928 | 52.44 [47.00; 60.00] | 4.14e-03 |
| **COPD** [% Yes] | 149486 | 2.83 | 50227 | 4.94 | 0.05 |
| **Age at diagnosis** [Years] | 4250 | 43.07 [32.00; 54.00] | 2483 | 46.14 [37.00; 57.00] | -1.08e-03 |
| **Anxiety** [% Yes] | 151792 | 6.98 | 50937 | 8.67 | -0.03 |
| **Age at diagnosis** [Years] | 10625 | 38.81 [29.00; 48.00] | 4414 | 41.65 [32.00; 51.00] | 1.70e-03 |
| **Depression** [% Yes] | 151137 | 13.37 | 50642 | 15.98 | -0.03 |
| **Age at diagnosis** [Years] | 20246 | 39.95 [30.00; 49.00] | 8094 | 42.11 [32.00; 52.00] | 0.01 |

**Table S2.** Associations between the mediators and the **presence of the diseases**.

|  |  | **Whole sample** | **Men** | **Women** | **Older cohort (born <=1970)** | **Younger cohort (born >1970)** |
| --- | --- | --- | --- | --- | --- | --- |
|  |  | **RR [95%-CI]** | **RR [95%-CI]** | **RR [95%-CI]** | **RR [95%-CI]** | **RR [95%-CI]** |
| **Ever Smoking** | **Any cancer** | 1.14 [1.08; 1.20] | 1.03 [0.95; 1.12] | 1.18 [1.11; 1.26] | 1.12 [1.06; 1.18] | 1.22 [1.02; 1.48] |
|  | **Smoking-rel. cancer** | 1.35 [1.25; 1.46] | 1.67 [1.39; 2.01] | 1.26 [1.16; 1.37] | 1.28 [1.19; 1.39] | 1.89 [1.41; 2.52] |
|  | **Obesity-rel. cancer** | 1.07 [1.00; 1.13] | 1.02 [0.93; 1.12] | 1.06 [0.98; 1.15] | 1.06 [1.00; 1.13] | 0.96 [0.75; 1.23] |
|  | **MI** | 2.00 [1.81; 2.21] | 2.05 [1.83; 2.29] | 1.85 [1.48; 2.32] | 1.97 [1.78; 2.18] | 2.47 [1.52; 4.00] |
|  | **Stroke** | 1.24 [1.13; 1.37] | 1.21 [1.07; 1.36] | 1.28 [1.11; 1.49] | 1.23 [1.12; 1.36] | 1.28 [0.95; 1.72] |
|  | **Diabetes** | 1.27 [1.20; 1.34] | 1.31 [1.23; 1.40] | 1.21 [1.11; 1.31] | 1.27 [1.20; 1.33] | 1.19 [0.96; 1.48] |
|  | **COPD** | 1.89 [1.77; 2.02] | 2.03 [1.83; 2.26] | 1.78 [1.64; 1.94] | 1.90 [1.77; 2.04] | 1.72 [1.44; 2.05] |
|  | **Anxiety** | 1.52 [1.46; 1.58] | 1.53 [1.43; 1.64] | 1.51 [1.44; 1.59] | 1.43 [1.37; 1.50] | 1.65 [1.54; 1.76] |
|  | **Depression** | 1.33 [1.30; 1.37] | 1.33 [1.27; 1.40] | 1.34 [1.30; 1.38] | 1.28 [1.24; 1.32] | 1.42 [1.36; 1.50] |
| **Current Smoking** | **Any cancer** | 1.69 [1.58; 1.80] | 1.46 [1.32; 1.62] | 1.81 [1.68; 1.96] | 1.63 [1.53; 1.74] | 1.86 [1.51; 2.28] |
|  | **Smoking-rel. cancer** | 2.18 [1.99; 2.38] | 2.73 [2.20; 3.38] | 2.03 [1.84; 2.25] | 2.03 [1.85; 2.23] | 3.06 [2.25; 4.17] |
|  | **Obesity-rel. cancer** | 1.44 [1.34; 1.56] | 1.35 [1.19; 1.52] | 1.47 [1.34; 1.63] | 1.43 [1.32; 1.55] | 1.25 [0.94; 1.65] |
|  | **MI** | 4.48 [4.02; 4.99] | 4.67 [4.14; 5.26] | 3.77 [2.92; 4.86] | 4.43 [3.97; 4.94] | 4.41 [2.64; 7.37] |
|  | **Stroke** | 2.15 [1.93; 2.41] | 2.10 [1.82; 2.43] | 2.18 [1.83; 2.60] | 2.16 [1.92; 2.44] | 2.07 [1.48; 2.90] |
|  | **Diabetes** | 1.71 [1.61; 1.82] | 1.80 [1.66; 1.95] | 1.57 [1.41; 1.75] | 1.69 [1.58; 1.81] | 1.75 [1.38; 2.22] |
|  | **COPD** | 3.99 [3.72; 4.28] | 4.44 [3.97; 4.96] | 3.67 [3.35; 4.02] | 4.10 [3.80; 4.42] | 3.14 [2.61; 3.77] |
|  | **Anxiety** | 2.50 [2.40; 2.61] | 2.53 [2.35; 2.73] | 2.48 [2.36; 2.61] | 2.47 [2.34; 2.60] | 2.49 [2.32; 2.68] |
|  | **Depression** | 2.10 [2.04; 2.16] | 2.16 [2.06; 2.28] | 2.06 [1.99; 2.14] | 2.06 [1.98; 2.13] | 2.13 [2.02; 2.24] |
| **Smoking Initiation Age** | **Any cancer** | 0.98 [0.98; 0.99] | 0.99 [0.99; 1.00] | 0.98 [0.97; 0.98] | 0.99 [0.98; 0.99] | 0.94 [0.90; 0.97] |
|  | **Smoking-rel. cancer** | 0.97 [0.96; 0.98] | 0.99 [0.97; 1.01] | 0.97 [0.96; 0.98] | 0.98 [0.97; 0.99] | 0.92 [0.87; 0.97] |
|  | **Obesity-rel. cancer** | 0.99 [0.98; 1.00] | 1.00 [0.99; 1.01] | 0.99 [0.98; 0.99] | 0.99 [0.99; 1.00] | 0.97 [0.93; 1.01] |
|  | **MI** | 0.99 [0.98; 1.00] | 0.99 [0.98; 1.00] | 1.02 [1.00; 1.04] | 0.99 [0.99; 1.00] | 0.98 [0.92; 1.04] |
|  | **Stroke** | 0.99 [0.99; 1.00] | 0.99 [0.98; 1.01] | 1.00 [0.98; 1.01] | 1.00 [0.99; 1.01] | 0.99 [0.95; 1.04] |
|  | **Diabetes** | 0.99 [0.99; 1.00] | 0.99 [0.99; 1.00] | 0.99 [0.98; 1.00] | 0.99 [0.99; 1.00] | 0.94 [0.90; 0.98] |
|  | **COPD** | 0.96 [0.95; 0.97] | 0.96 [0.95; 0.97] | 0.95 [0.94; 0.97] | 0.96 [0.95; 0.97] | 0.92 [0.88; 0.95] |
|  | **Anxiety** | 0.98 [0.97; 0.98] | 0.98 [0.97; 0.99] | 0.98 [0.97; 0.98] | 0.99 [0.98; 0.99] | 0.95 [0.94; 0.96] |
|  | **Depression** | 0.98 [0.97; 0.98] | 0.98 [0.97; 0.98] | 0.98 [0.97; 0.98] | 0.98 [0.98; 0.98] | 0.96 [0.96; 0.97] |

*rel.=related; MI=myocardial infarction; diabetes=type 2 diabetes; COPD=chronic bronchitis or chronic obstructive pulmonary disease; Anxiety=anxiety disorder or panic attacks; RR=risk ratio; 95%‑CI=95% confidence interval*

**Table S3.** Mediation analyses between **childhood abuse** and the presence of the diseases.

|  |  | **Whole sample** | | | **Men** | | | **Women** | | | **Older cohort (born <=1970)** | | | **Younger cohort (born >1970)** | | |
| --- | --- | --- | --- | --- | --- | --- | --- | --- | --- | --- | --- | --- | --- | --- | --- | --- |
|  |  | **PDE** | **TIE** |  | **PDE** | **TIE** |  | **PDE** | **TIE** |  | **PDE** | **TIE** |  | **PDE** | **TIE** |  |
|  |  | **RR [95%-CI]** | **RR [95%-CI]** | **% [95%-CI]** | **RR [95%-CI]** | **RR [95%-CI]** | **% [95%-CI]** | **RR [95%-CI]** | **RR [95%-CI]** | **% [95%-CI]** | **RR [95%-CI]** | **RR [95%-CI]** | **% [95%-CI]** | **RR [95%-CI]** | **RR [95%-CI]** | **% [95%-CI]** |
| **Ever Smoking** | **Any cancer** | 1.14 [1.07; 1.22] | 1.02 [1.01; 1.03] | **13.90 [2.33; 25.47]** | 1.03 [0.91; 1.16] | 1.01 [0.98; 1.04] | **30.23 [-87.30; 147.77]** | 1.19 [1.09; 1.29] | 1.02 [1.01; 1.04] | **13.38 [1.62; 25.14]** | 1.11 [1.03; 1.19] | 1.02 [1.00; 1.03] | **13.59 [-1.62; 28.80]** | 1.48 [1.18; 1.85] | 1.05 [1.00; 1.10] | **13.71 [0.12; 27.31]** |
|  | **Smoking-rel. cancer** | 1.19 [1.09; 1.31] | 1.04 [1.02; 1.06] | **19.07 [6.97; 31.16]** | 0.98 [0.77; 1.23] | 1.10 [1.05; 1.16] | **131.83 [-257.61; 521.26]** | 1.23 [1.10; 1.36] | 1.03 [1.01; 1.05] | **13.87 [1.16; 26.57]** | 1.15 [1.04; 1.27] | 1.03 [1.01; 1.05] | **19.13 [3.51; 34.75]** | 1.57 [1.14; 2.17] | 1.08 [1.01; 1.15] | **18.07 [1.89; 34.25]** |
|  | **Obesity-rel. cancer** | 1.08 [1.00; 1.17] | 1.00 [0.99; 1.02] | **5.88 [-17.20; 28.96]** | 1.02 [0.89; 1.18] | 1.01 [0.98; 1.04] | **26.03 [-144.18; 196.24]** | 1.10 [1.00; 1.22] | 1.00 [0.98; 1.02] | **2.07 [-20.69; 24.84]** | 1.06 [0.98; 1.15] | 1.00 [0.98; 1.02] | **1.15 [-29.73; 32.02]** | 1.25 [0.92; 1.71] | 1.04 [0.97; 1.11] | **16.90 [-15.21; 49.01]** |
|  | **MI** | 1.18 [1.05; 1.32] | 1.09 [1.06; 1.12] | **36.88 [17.88; 55.89]** | 1.11 [0.97; 1.27] | 1.09 [1.06; 1.12] | **48.62 [14.28; 82.97]** | 1.43 [1.11; 1.85] | 1.08 [1.02; 1.14] | **20.82 [2.75; 38.89]** | 1.17 [1.04; 1.32] | 1.08 [1.05; 1.11] | **35.75 [16.04; 55.46]** | 1.15 [0.65; 2.03] | 1.14 [1.02; 1.28] | **51.80 [-47.62; 151.22]** |
|  | **Stroke** | 1.43 [1.28; 1.60] | 1.03 [1.00; 1.05] | **8.48 [0.61; 16.34]** | 1.35 [1.15; 1.58] | 1.02 [0.98; 1.05] | **5.89 [-7.18; 18.96]** | 1.53 [1.29; 1.83] | 1.04 [1.00; 1.08] | **11.00 [0.59; 21.42]** | 1.43 [1.27; 1.60] | 1.02 [0.99; 1.04] | **5.79 [-2.37; 13.95]** | 1.47 [1.02; 2.10] | 1.11 [1.03; 1.19] | **25.92 [3.85; 47.99]** |
|  | **Diabetes** | 1.31 [1.22; 1.39] | 1.02 [1.01; 1.04] | **8.62 [2.62; 14.63]** | 1.32 [1.21; 1.44] | 1.02 [1.00; 1.04] | **8.16 [0.41; 15.90]** | 1.29 [1.16; 1.43] | 1.02 [1.00; 1.05] | **9.14 [-0.96; 19.24]** | 1.27 [1.18; 1.36] | 1.02 [1.01; 1.04] | **9.48 [2.77; 16.20]** | 1.85 [1.44; 2.38] | 1.01 [0.95; 1.06] | **1.51 [-9.89; 12.91]** |
|  | **COPD** | 1.62 [1.51; 1.73] | 1.07 [1.06; 1.09] | **15.56 [12.04; 19.08]** | 1.54 [1.38; 1.72] | 1.08 [1.05; 1.10] | **17.75 [11.14; 24.35]** | 1.66 [1.51; 1.83] | 1.07 [1.05; 1.10] | **15.63 [10.75; 20.51]** | 1.57 [1.46; 1.69] | 1.06 [1.05; 1.08] | **15.10 [11.24; 18.96]** | 1.88 [1.55; 2.28] | 1.08 [1.04; 1.13] | **15.36 [7.72; 23.01]** |
|  | **Anxiety** | 2.11 [2.03; 2.20] | 1.03 [1.02; 1.04] | **5.61 [4.09; 7.13]** | 2.29 [2.12; 2.46] | 1.03 [1.02; 1.05] | **5.59 [3.01; 8.18]** | 2.07 [1.96; 2.18] | 1.03 [1.02; 1.04] | **5.65 [3.60; 7.69]** | 1.99 [1.89; 2.09] | 1.03 [1.02; 1.04] | **6.17 [4.27; 8.08]** | 2.35 [2.18; 2.53] | 1.03 [1.01; 1.04] | **4.50 [2.02; 6.98]** |
|  | **Depression** | 2.22 [2.15; 2.29] | 1.02 [1.01; 1.02] | **2.66 [1.61; 3.71]** | 2.30 [2.18; 2.43] | 1.02 [1.01; 1.03] | **2.73 [0.89; 4.57]** | 2.22 [2.13; 2.30] | 1.01 [1.01; 1.02] | **2.62 [1.28; 3.95]** | 2.08 [2.01; 2.16] | 1.01 [1.01; 1.02] | **2.72 [1.42; 4.02]** | 2.51 [2.38; 2.66] | 1.02 [1.01; 1.03] | **2.68 [0.96; 4.40]** |

*Regression and mediation models were adjusted for age at NAKO baseline examination, study center, and educational years. Sex was used as a covariate for the whole sample and the birth cohort subgroups. Smoking status was defined before disease diagnosis. PDE=pure direct effect; TIE=total indirect effect; rel.=related; MI=myocardial infarction; diabetes=type 2 diabetes; COPD=chronic bronchitis or chronic obstructive pulmonary disease; Anxiety=anxiety disorder or panic attacks; RR=risk ratio; 95%-CI=95% confidence interval; %=proportion mediated*

**Table S4.** Associations between the mediators and the **presence of the diseases**.

|  |  | **Whole sample** | **Men** | **Women** | **Older cohort (born <=1970)** | **Younger cohort (born >1970)** |
| --- | --- | --- | --- | --- | --- | --- |
|  |  | **RR [95%-CI]** | **RR [95%-CI]** | **RR [95%-CI]** | **RR [95%-CI]** | **RR [95%-CI]** |
| **Early-onset Overweight** | **Any cancer** | 1.08 [1.01; 1.16] | 1.06 [0.96; 1.17] | 1.10 [1.00; 1.20] | 1.06 [0.99; 1.14] | 1.31 [1.04; 1.64] |
|  | **Smoking-rel. cancer** | 1.13 [1.02; 1.25] | 1.30 [1.08; 1.58] | 1.07 [0.95; 1.20] | 1.12 [1.01; 1.25] | 1.21 [0.85; 1.74] |
|  | **Obesity-rel. cancer** | 1.07 [0.98; 1.16] | 1.09 [0.97; 1.22] | 1.04 [0.93; 1.16] | 1.05 [0.97; 1.14] | 1.29 [0.95; 1.76] |
|  | **MI** | 1.34 [1.21; 1.49] | 1.25 [1.12; 1.40] | 1.94 [1.51; 2.50] | 1.31 [1.18; 1.46] | 2.14 [1.37; 3.33] |
|  | **Stroke** | 1.24 [1.11; 1.39] | 1.09 [0.95; 1.26] | 1.55 [1.29; 1.86] | 1.23 [1.09; 1.39] | 1.29 [0.91; 1.83] |
|  | **Diabetes** | 2.16 [2.05; 2.28] | 2.01 [1.89; 2.14] | 2.48 [2.27; 2.72] | 2.04 [1.93; 2.15] | 4.86 [3.93; 6.01] |
|  | **COPD** | 1.51 [1.41; 1.63] | 1.36 [1.23; 1.50] | 1.69 [1.54; 1.87] | 1.46 [1.35; 1.58] | 1.89 [1.57; 2.27] |
|  | **Anxiety** | 1.37 [1.31; 1.43] | 1.20 [1.12; 1.30] | 1.49 [1.41; 1.57] | 1.36 [1.28; 1.43] | 1.41 [1.30; 1.52] |
|  | **Depression** | 1.45 [1.40; 1.49] | 1.27 [1.21; 1.33] | 1.58 [1.52; 1.64] | 1.40 [1.35; 1.45] | 1.55 [1.47; 1.64] |
| **Early-onset Overweight 18+** | **Any cancer** | 1.07 [0.97; 1.17] | 1.02 [0.90; 1.16] | 1.10 [0.96; 1.26] | 1.05 [0.95; 1.16] | 1.37 [1.03; 1.83] |
|  | **Smoking-rel. cancer** | 1.18 [1.03; 1.36] | 1.33 [1.06; 1.69] | 1.10 [0.92; 1.30] | 1.16 [1.00; 1.35] | 1.56 [1.01; 2.39] |
|  | **Obesity-rel. cancer** | 1.03 [0.92; 1.15] | 1.03 [0.89; 1.20] | 1.00 [0.85; 1.18] | 1.03 [0.92; 1.16] | 1.13 [0.74; 1.72] |
|  | **MI** | 1.28 [1.12; 1.46] | 1.21 [1.05; 1.39] | 1.92 [1.35; 2.71] | 1.26 [1.10; 1.44] | 2.03 [1.20; 3.41] |
|  | **Stroke** | 1.15 [0.99; 1.34] | 1.02 [0.85; 1.22] | 1.55 [1.19; 2.01] | 1.19 [1.01; 1.39] | 0.92 [0.54; 1.54] |
|  | **Diabetes** | 2.35 [2.21; 2.50] | 2.09 [1.94; 2.25] | 3.14 [2.81; 3.50] | 2.21 [2.07; 2.36] | 5.46 [4.36; 6.83] |
|  | **COPD** | 1.51 [1.38; 1.66] | 1.28 [1.12; 1.45] | 1.87 [1.64; 2.14] | 1.47 [1.33; 1.63] | 1.84 [1.46; 2.32] |
|  | **Anxiety** | 1.26 [1.18; 1.34] | 1.11 [1.00; 1.22] | 1.40 [1.29; 1.52] | 1.25 [1.15; 1.35] | 1.33 [1.21; 1.47] |
|  | **Depression** | 1.37 [1.31; 1.43] | 1.15 [1.07; 1.22] | 1.59 [1.51; 1.68] | 1.33 [1.26; 1.40] | 1.51 [1.41; 1.61] |

*rel.=related; MI=myocardial infarction; diabetes=type 2 diabetes; COPD=chronic bronchitis or chronic obstructive pulmonary disease; Anxiety=anxiety disorder or panic attacks; RR=risk ratio; 95%‑CI=95% confidence interval*

**Table S5.** Mediation analyses between **childhood abuse** and the presence of the diseases.

|  |  | **Whole sample** | | | **Men** | | | **Women** | | | **Older cohort (born <=1970)** | | | **Younger cohort (born >1970)** | | |
| --- | --- | --- | --- | --- | --- | --- | --- | --- | --- | --- | --- | --- | --- | --- | --- | --- |
|  |  | **PDE** | **TIE** |  | **PDE** | **TIE** |  | **PDE** | **TIE** |  | **PDE** | **TIE** |  | **PDE** | **TIE** |  |
|  |  | **RR [95%-CI]** | **RR [95%-CI]** | **% [95%-CI]** | **RR [95%-CI]** | **RR [95%-CI]** | **% [95%-CI]** | **RR [95%-CI]** | **RR [95%-CI]** | **% [95%-CI]** | **RR [95%-CI]** | **RR [95%-CI]** | **% [95%-CI]** | **RR [95%-CI]** | **RR [95%-CI]** | **% [95%-CI]** |
| **Early-onset overweight** | **Any cancer** | 1.16 [1.09; 1.24] | 1.00 [1.00; 1.01] | **2.98 [-1.50; 7.45]** | 1.05 [0.93; 1.17] | 1.01 [1.00; 1.01] | **13.07 [-17.01; 43.15]** | 1.22 [1.12; 1.32] | 1.00 [0.99; 1.01] | **0.64 [-3.77; 5.06]** | 1.12 [1.05; 1.20] | 1.00 [1.00; 1.01] | **1.68 [-3.55; 6.91]** | 1.55 [1.24; 1.93] | 1.03 [1.00; 1.06] | **7.25 [-0.01; 14.51]** |
|  | **Smoking-rel. cancer** | 1.22 [1.11; 1.34] | 1.00 [0.99; 1.01] | **-0.25 [-4.99; 4.49]** | 1.09 [0.88; 1.36] | 1.01 [1.00; 1.03] | **11.45 [-16.74; 39.63]** | 1.26 [1.14; 1.40] | 1.00 [0.99; 1.01] | **-2.09 [-6.82; 2.64]** | 1.17 [1.06; 1.29] | 1.00 [0.99; 1.01] | **-1.12 [-6.50; 4.26]** | 1.69 [1.23; 2.32] | 1.01 [0.98; 1.05] | **3.38 [-5.08; 11.84]** |
|  | **Obesity-rel. cancer** | 1.08 [1.00; 1.17] | 1.01 [1.00; 1.01] | **6.51 [-3.47; 16.49]** | 1.04 [0.91; 1.18] | 1.01 [1.00; 1.02] | **21.68 [-42.74; 86.10]** | 1.10 [1.00; 1.21] | 1.00 [0.99; 1.01] | **0.30 [-9.91; 10.50]** | 1.06 [0.98; 1.15] | 1.00 [1.00; 1.01] | **4.77 [-7.44; 16.97]** | 1.31 [0.96; 1.78] | 1.04 [1.00; 1.08] | **14.58 [-2.76; 31.91]** |
|  | **MI** | 1.25 [1.12; 1.40] | 1.01 [1.00; 1.02] | **4.16 [-1.17; 9.50]** | 1.19 [1.05; 1.35] | 1.00 [0.99; 1.01] | **1.07 [-3.78; 5.92]** | 1.49 [1.17; 1.90] | 1.03 [1.00; 1.06] | **8.96 [0.34; 17.59]** | 1.24 [1.11; 1.39] | 1.00 [1.00; 1.01] | **2.41 [-2.49; 7.30]** | 1.29 [0.74; 2.23] | 1.10 [1.03; 1.18] | **31.02 [-13.36; 75.41]** |
|  | **Stroke** | 1.46 [1.31; 1.63] | 1.01 [1.00; 1.03] | **4.39 [1.05; 7.72]** | 1.36 [1.17; 1.57] | 1.00 [0.99; 1.01] | **0.67 [-2.68; 4.02]** | 1.57 [1.33; 1.86] | 1.03 [1.01; 1.05] | **7.91 [2.66; 13.16]** | 1.44 [1.28; 1.62] | 1.01 [1.00; 1.02] | **3.70 [0.41; 7.00]** | 1.63 [1.15; 2.32] | 1.03 [0.99; 1.07] | **6.97 [-3.24; 17.17]** |
|  | **Diabetes** | 1.29 [1.21; 1.38] | 1.04 [1.03; 1.04] | **13.69 [9.85; 17.52]** | 1.31 [1.21; 1.42] | 1.02 [1.01; 1.03] | **8.43 [4.52; 12.35]** | 1.27 [1.15; 1.40] | 1.04 [1.03; 1.06] | **16.16 [8.92; 23.39]** | 1.26 [1.18; 1.34] | 1.03 [1.02; 1.04] | **11.76 [7.58; 15.95]** | 1.72 [1.35; 2.19] | 1.11 [1.07; 1.15] | **20.63 [12.67; 28.59]** |
|  | **COPD** | 1.70 [1.59; 1.82] | 1.02 [1.01; 1.03] | **4.73 [3.03; 6.43]** | 1.63 [1.47; 1.81] | 1.01 [1.01; 1.02] | **3.41 [1.30; 5.52]** | 1.74 [1.59; 1.91] | 1.02 [1.01; 1.03] | **4.89 [2.59; 7.18]** | 1.65 [1.53; 1.77] | 1.02 [1.01; 1.02] | **4.00 [2.23; 5.78]** | 1.98 [1.63; 2.39] | 1.04 [1.01; 1.06] | **6.97 [2.62; 11.32]** |
|  | **Anxiety** | 2.15 [2.06; 2.24] | 1.01 [1.00; 1.01] | **1.60 [0.84; 2.35]** | 2.34 [2.17; 2.51] | 1.00 [1.00; 1.01] | **0.36 [-0.41; 1.13]** | 2.08 [1.98; 2.19] | 1.01 [1.01; 1.02] | **2.21 [1.18; 3.23]** | 2.03 [1.93; 2.13] | 1.01 [1.00; 1.01] | **1.31 [0.46; 2.17]** | 2.38 [2.21; 2.56] | 1.01 [1.00; 1.02] | **2.16 [0.70; 3.62]** |
|  | **Depression** | 2.22 [2.15; 2.28] | 1.01 [1.01; 1.01] | **2.03 [1.43; 2.62]** | 2.31 [2.19; 2.43] | 1.00 [1.00; 1.01] | **0.56 [-0.02; 1.15]** | 2.19 [2.11; 2.27] | 1.02 [1.01; 1.02] | **2.79 [1.96; 3.62]** | 2.08 [2.01; 2.16] | 1.01 [1.00; 1.01] | **1.60 [0.94; 2.26]** | 2.50 [2.37; 2.64] | 1.02 [1.01; 1.02] | **2.81 [1.64; 3.98]** |

*Regression and mediation models were adjusted for age at NAKO baseline examination, study center, and educational years. Sex was used as a covariate for the whole sample and the birth cohort subgroups. Smoking status was defined before disease diagnosis. PDE=pure direct effect; TIE=total indirect effect; MI=myocardial infarction; diabetes=type 2 diabetes; COPD=chronic bronchitis or chronic obstructive pulmonary disease; Anxiety=anxiety disorder or panic attacks; RR=risk ratio; 95%-CI=95% confidence interval; %=proportion mediated*

**Table S6.** Mediation analyses between childhood maltreatment and the presence of the diseases via **current smoking**.

|  |  | **Whole sample** | | | **Men** | | | **Women** | | | **Older cohort (born <=1970)** | | | **Younger cohort (born >1970)** | | |
| --- | --- | --- | --- | --- | --- | --- | --- | --- | --- | --- | --- | --- | --- | --- | --- | --- |
|  |  | **PDE** | **TIE** |  | **PDE** | **TIE** |  | **PDE** | **TIE** |  | **PDE** | **TIE** |  | **PDE** | **TIE** |  |
|  |  | **RR [95%-CI]** | **RR [95%-CI]** | **% [95%-CI]** | **RR [95%-CI]** | **RR [95%-CI]** | **% [95%-CI]** | **RR [95%-CI]** | **RR [95%-CI]** | **% [95%-CI]** | **RR [95%-CI]** | **RR [95%-CI]** | **% [95%-CI]** | **RR [95%-CI]** | **RR [95%-CI]** | **% [95%-CI]** |
| **Abuse** | **Any cancer** | 1.13 [1.04; 1.22] | 1.08 [1.06; 1.11] | **42.52 [24.86; 60.17]** | 1.03 [0.89; 1.20] | 1.07 [1.03; 1.11] | **68.10 [-33.78; 169.98]** | 1.16 [1.05; 1.28] | 1.08 [1.05; 1.11] | **36.94 [18.61; 55.26]** | 1.09 [1.00; 1.18] | 1.07 [1.05; 1.09] | **46.32 [18.75; 73.89]** | 1.43 [1.12; 1.83] | 1.13 [1.06; 1.20] | **29.37 [11.70; 47.05]** |
|  | **Smoking-rel. cancer** | 1.14 [1.02; 1.28] | 1.11 [1.08; 1.15] | **47.57 [26.27; 68.88]** | 0.85 [0.62; 1.16] | 1.21 [1.11; 1.31] | **627.00** | 1.20 [1.06; 1.35] | 1.09 [1.06; 1.13] | **36.39 [17.54; 55.24]** | 1.09 [0.97; 1.23] | 1.09 [1.06; 1.13] | **52.31 [17.93; 86.70]** | 1.57 [1.11; 2.22] | 1.17 [1.08; 1.27] | **32.51 [13.38; 51.64]** |
|  | **Obesity-rel. cancer** | 1.07 [0.97; 1.18] | 1.05 [1.02; 1.08] | **41.28 [5.24; 77.33]** | 1.04 [0.87; 1.25] | 1.06 [1.01; 1.11] | **58.50 [-49.53; 166.53]** | 1.08 [0.96; 1.22] | 1.04 [1.01; 1.07] | **31.24 [-7.63; 70.11]** | 1.06 [0.96; 1.18] | 1.04 [1.01; 1.07] | **40.26 [-5.86; 86.37]** | 1.15 [0.80; 1.64] | 1.08 [0.98; 1.18] | **38.47 [-31.18; 108.11]** |
|  | **MI** | 1.17 [1.03; 1.34] | 1.25 [1.21; 1.29] | **62.71 [43.61; 81.80]** | 1.08 [0.93; 1.26] | 1.25 [1.20; 1.30] | **76.40 [42.00; 110.80]** | 1.52 [1.14; 2.01] | 1.23 [1.14; 1.32] | **39.81 [21.68; 57.94]** | 1.16 [1.01; 1.34] | 1.24 [1.20; 1.29] | **63.41 [42.08; 84.74]** | 1.14 [0.62; 2.08] | 1.27 [1.11; 1.45] | **68.88 [-28.70; 166.46]** |
|  | **Stroke** | 1.38 [1.20; 1.57] | 1.11 [1.07; 1.15] | **29.42 [17.94; 40.89]** | 1.24 [1.02; 1.51] | 1.08 [1.03; 1.13] | **28.82 [4.50; 53.15]** | 1.53 [1.26; 1.87] | 1.14 [1.08; 1.20] | **28.17 [15.07; 41.26]** | 1.40 [1.21; 1.62] | 1.10 [1.06; 1.14] | **25.96 [14.04; 37.87]** | 1.21 [0.80; 1.83] | 1.19 [1.07; 1.32] | **52.05 [-1.69; 105.79]** |
|  | **Diabetes** | 1.30 [1.19; 1.41] | 1.07 [1.05; 1.09] | **23.65 [15.02; 32.28]** | 1.28 [1.14; 1.43] | 1.06 [1.03; 1.09] | **21.94 [9.39; 34.50]** | 1.33 [1.17; 1.51] | 1.07 [1.04; 1.11] | **22.78 [10.86; 34.70]** | 1.26 [1.15; 1.37] | 1.07 [1.04; 1.09] | **24.39 [13.93; 34.84]** | 1.79 [1.36; 2.36] | 1.05 [0.99; 1.13] | **10.97 [-3.11; 25.05]** |
|  | **COPD** | 1.57 [1.45; 1.70] | 1.21 [1.19; 1.24] | **36.81 [32.12; 41.50]** | 1.51 [1.33; 1.71] | 1.22 [1.18; 1.26] | **39.50 [30.95; 48.04]** | 1.61 [1.45; 1.79] | 1.20 [1.16; 1.24] | **34.60 [28.19; 41.00]** | 1.54 [1.41; 1.68] | 1.21 [1.18; 1.24] | **37.41 [31.75; 43.08]** | 1.74 [1.42; 2.13] | 1.19 [1.13; 1.25] | **30.68 [20.93; 40.43]** |
|  | **Anxiety** | 2.09 [1.99; 2.20] | 1.12 [1.10; 1.13] | **18.39 [16.21; 20.57]** | 2.22 [2.03; 2.42] | 1.11 [1.09; 1.14] | **17.28 [13.52; 21.05]** | 2.07 [1.95; 2.20] | 1.11 [1.09; 1.13] | **17.44 [14.66; 20.22]** | 1.96 [1.85; 2.09] | 1.12 [1.11; 1.14] | **20.30 [17.32; 23.28]** | 2.30 [2.13; 2.49] | 1.09 [1.07; 1.11] | **13.68 [10.47; 16.89]** |
|  | **Depression** | 2.16 [2.09; 2.24] | 1.08 [1.07; 1.09] | **13.35 [11.72; 14.97]** | 2.23 [2.10; 2.38] | 1.09 [1.07; 1.11] | **13.66 [10.85; 16.48]** | 2.17 [2.08; 2.27] | 1.07 [1.06; 1.09] | **11.86 [9.90; 13.82]** | 2.03 [1.95; 2.12] | 1.08 [1.07; 1.10] | **13.96 [11.82; 16.09]** | 2.41 [2.27; 2.56] | 1.07 [1.05; 1.09] | **10.74 [8.31; 13.18]** |
| **Neglect** | **Any cancer** | 1.08 [1.00; 1.17] | 1.05 [1.03; 1.06] | **39.34 [14.92; 63.75]** | 1.09 [0.96; 1.24] | 1.03 [1.02; 1.05] | **28.89 [-1.89; 59.66]** | 1.07 [0.97; 1.18] | 1.05 [1.03; 1.07] | **43.69 [5.64; 81.74]** | 1.07 [0.98; 1.16] | 1.04 [1.03; 1.05] | **39.10 [7.81; 70.38]** | 1.29 [0.97; 1.73] | 1.09 [1.03; 1.15] | **28.42 [2.44; 54.40]** |
|  | **Smoking-rel. cancer** | 1.08 [0.96; 1.20] | 1.07 [1.05; 1.09] | **49.92 [12.23; 87.60]** | 1.22 [0.96; 1.57] | 1.06 [1.03; 1.09] | **24.03 [0.49; 47.57]** | 1.05 [0.93; 1.19] | 1.07 [1.04; 1.10] | **60.19 [-0.90; 121.28]** | 1.04 [0.93; 1.17] | 1.05 [1.03; 1.08] | **56.64 [-10.11; 123.39]** | 1.63 [1.11; 2.41] | 1.15 [1.08; 1.23] | **27.71 [10.86; 44.57]** |
|  | **Obesity-rel. cancer** | 1.00 [0.91; 1.09] | 1.03 [1.01; 1.05] | **115.77 [-302.39; 533.93]** | 0.99 [0.85; 1.16] | 1.03 [1.01; 1.06] | **132.27 [-658.82; 923.36]** | 1.00 [0.89; 1.13] | 1.02 [0.99; 1.04] | **79.98 [-380.48; 540.45]** | 1.00 [0.91; 1.10] | 1.03 [1.01; 1.04] | **98.29 [-271.44; 468.01]** | 0.92 [0.58; 1.44] | 1.05 [0.96; 1.14] | **-100.22** |
|  | **MI** | 0.92 [0.81; 1.05] | 1.14 [1.11; 1.16] | **252.88 [-398.71; 904.46]** | 0.83 [0.72; 0.97] | 1.09 [1.06; 1.12] | **-85.15 [-229.51; 59.20]** | 1.38 [1.05; 1.82] | 1.18 [1.11; 1.26] | **39.73 [18.47; 60.98]** | 0.92 [0.80; 1.05] | 1.13 [1.10; 1.16] | **341.05** | 1.36 [0.74; 2.47] | 1.15 [1.03; 1.27] | **35.81 [-10.48; 82.10]** |
|  | **Stroke** | 1.33 [1.17; 1.51] | 1.07 [1.04; 1.09] | **21.76 [12.47; 31.05]** | 1.23 [1.04; 1.46] | 1.05 [1.03; 1.07] | **21.31 [5.92; 36.69]** | 1.49 [1.22; 1.81] | 1.07 [1.03; 1.12] | **17.77 [5.93; 29.61]** | 1.29 [1.13; 1.48] | 1.06 [1.04; 1.09] | **21.89 [10.68; 33.10]** | 1.89 [1.26; 2.83] | 1.08 [1.00; 1.16] | **14.42 [0.26; 28.58]** |
|  | **Diabetes** | 1.09 [1.01; 1.18] | 1.04 [1.02; 1.05] | **32.09 [10.82; 53.36]** | 1.08 [0.97; 1.19] | 1.02 [1.01; 1.04] | **23.98 [-3.74; 51.70]** | 1.12 [0.99; 1.27] | 1.06 [1.03; 1.08] | **33.73 [6.53; 60.93]** | 1.08 [1.00; 1.17] | 1.03 [1.02; 1.05] | **31.82 [6.84; 56.80]** | 1.41 [1.03; 1.92] | 1.04 [0.98; 1.10] | **10.86 [-8.06; 29.77]** |
|  | **COPD** | 1.30 [1.20; 1.41] | 1.13 [1.11; 1.15] | **35.92 [28.61; 43.23]** | 1.14 [1.00; 1.29] | 1.09 [1.07; 1.12] | **44.02 [19.71; 68.32]** | 1.45 [1.31; 1.62] | 1.15 [1.12; 1.19] | **33.16 [25.52; 40.80]** | 1.27 [1.17; 1.39] | 1.12 [1.10; 1.15] | **36.70 [27.84; 45.56]** | 1.59 [1.27; 2.00] | 1.13 [1.08; 1.18] | **25.69 [14.41; 36.97]** |
|  | **Anxiety** | 1.63 [1.55; 1.71] | 1.08 [1.06; 1.09] | **16.44 [13.84; 19.04]** | 1.60 [1.46; 1.75] | 1.07 [1.05; 1.08] | **15.13 [10.94; 19.32]** | 1.68 [1.57; 1.80] | 1.08 [1.06; 1.09] | **16.15 [12.69; 19.62]** | 1.60 [1.51; 1.71] | 1.08 [1.06; 1.09] | **16.73 [13.53; 19.93]** | 1.71 [1.55; 1.88] | 1.06 [1.04; 1.08] | **12.67 [8.17; 17.16]** |
|  | **Depression** | 1.65 [1.59; 1.71] | 1.06 [1.05; 1.07] | **12.56 [10.67; 14.44]** | 1.58 [1.48; 1.68] | 1.05 [1.04; 1.06] | **11.69 [8.63; 14.76]** | 1.72 [1.64; 1.81] | 1.06 [1.05; 1.07] | **12.13 [9.67; 14.58]** | 1.59 [1.53; 1.67] | 1.06 [1.05; 1.07] | **12.96 [10.62; 15.30]** | 1.84 [1.71; 1.98] | 1.04 [1.03; 1.06] | **8.76 [5.74; 11.78]** |

*The analyses compared never to current smokers prior to the diagnosis by excluding participants who both started and quit smoking before the respective diagnosis. Regression and mediation models were adjusted for age at NAKO baseline examination, study center, and educational years. Sex was used as a covariate for the whole sample and the birth cohort subgroups. Smoking status was defined before disease diagnosis. PDE=pure direct effect; TIE=total indirect effect; rel.=related; MI=myocardial infarction; diabetes=type 2 diabetes; COPD=chronic bronchitis or chronic obstructive pulmonary disease; Anxiety=anxiety disorder or panic attacks; RR=risk ratio; 95%-CI=95% confidence interval; %=proportion mediated*

**Table S7.** Mediation analyses between childhood maltreatment and the presence of the diseases via **early-onset overweight during adulthood (18+)**.

|  |  | **Whole sample** | | | **Men** | | | **Women** | | | **Older cohort (born <=1970)** | | | **Younger cohort (born >1970)** | | |
| --- | --- | --- | --- | --- | --- | --- | --- | --- | --- | --- | --- | --- | --- | --- | --- | --- |
|  |  | **PDE** | **TIE** |  | **PDE** | **TIE** |  | **PDE** | **TIE** |  | **PDE** | **TIE** |  | **PDE** | **TIE** |  |
|  |  | **RR [95%-CI]** | **RR [95%-CI]** | **% [95%-CI]** | **RR [95%-CI]** | **RR [95%-CI]** | **% [95%-CI]** | **RR [95%-CI]** | **RR [95%-CI]** | **% [95%-CI]** | **RR [95%-CI]** | **RR [95%-CI]** | **% [95%-CI]** | **RR [95%-CI]** | **RR [95%-CI]** | **% [95%-CI]** |
| **Abuse** | **Any cancer** | 1.15 [1.07; 1.23] | 1.00 [1.00; 1.01] | **1.31 [-2.59; 5.20]** | 1.02 [0.91; 1.15] | 1.00 [1.00; 1.01] | **7.90 [-37.23; 53.04]** | 1.21 [1.12; 1.32] | 1.00 [0.99; 1.01] | **0.34 [-3.05; 3.72]** | 1.11 [1.03; 1.19] | 1.00 [1.00; 1.00] | **-0.12 [-4.33; 4.08]** | 1.51 [1.19; 1.90] | 1.02 [1.00; 1.05] | **6.61 [-0.81; 14.04]** |
|  | **Smoking-rel. cancer** | 1.24 [1.13; 1.36] | 1.00 [0.99; 1.01] | **0.83 [-2.88; 4.53]** | 1.08 [0.87; 1.36] | 1.01 [1.00; 1.02] | **7.65 [-13.93; 29.24]** | 1.28 [1.16; 1.42] | 1.00 [0.99; 1.01] | **-0.68 [-3.94; 2.58]** | 1.18 [1.07; 1.31] | 1.00 [0.99; 1.01] | **0.01 [-3.77; 3.79]** | 1.70 [1.23; 2.36] | 1.02 [0.98; 1.06] | **4.55 [-3.37; 12.46]** |
|  | **Obesity-rel. cancer** | 1.08 [0.99; 1.17] | 1.00 [1.00; 1.01] | **2.55 [-5.87; 10.97]** | 1.01 [0.88; 1.16] | 1.00 [1.00; 1.01] | **18.21 [-140.37; 176.80]** | 1.10 [1.00; 1.22] | 1.00 [0.99; 1.01] | **-1.07 [-8.90; 6.75]** | 1.06 [0.97; 1.15] | 1.00 [1.00; 1.01] | **1.43 [-7.71; 10.56]** | 1.23 [0.88; 1.70] | 1.02 [0.98; 1.06] | **9.61 [-10.22; 29.44]** |
|  | **MI** | 1.24 [1.10; 1.39] | 1.00 [0.99; 1.01] | **0.37 [-3.66; 4.40]** | 1.19 [1.05; 1.36] | 1.00 [0.99; 1.00] | **-0.51 [-3.23; 2.21]** | 1.43 [1.11; 1.85] | 1.01 [0.99; 1.03] | **3.73 [-3.00; 10.46]** | 1.23 [1.10; 1.38] | 1.00 [0.99; 1.01] | **-0.46 [-3.73; 2.81]** | 1.20 [0.67; 2.15] | 1.07 [1.00; 1.15] | **29.59 [-32.62; 91.80]** |
|  | **Stroke** | 1.48 [1.33; 1.66] | 1.01 [1.00; 1.02] | **3.08 [0.42; 5.73]** | 1.38 [1.19; 1.60] | 1.00 [1.00; 1.01] | **0.77 [-1.17; 2.71]** | 1.59 [1.34; 1.88] | 1.02 [1.00; 1.04] | **5.02 [0.80; 9.24]** | 1.46 [1.30; 1.64] | 1.01 [1.00; 1.02] | **2.79 [0.26; 5.32]** | 1.64 [1.13; 2.38] | 1.00 [0.97; 1.04] | **1.03 [-8.10; 10.15]** |
|  | **Diabetes** | 1.31 [1.23; 1.40] | 1.03 [1.02; 1.03] | **9.91 [6.48; 13.35]** | 1.33 [1.22; 1.44] | 1.01 [1.00; 1.02] | **3.94 [0.89; 6.99]** | 1.28 [1.16; 1.42] | 1.04 [1.02; 1.05] | **14.20 [7.54; 20.86]** | 1.28 [1.20; 1.37] | 1.02 [1.01; 1.03] | **7.67 [4.02; 11.31]** | 1.70 [1.32; 2.19] | 1.10 [1.06; 1.14] | **19.50 [10.91; 28.08]** |
|  | **COPD** | 1.72 [1.60; 1.85] | 1.02 [1.01; 1.02] | **3.49 [1.95; 5.02]** | 1.61 [1.44; 1.80] | 1.01 [1.00; 1.01] | **1.65 [0.12; 3.17]** | 1.77 [1.61; 1.94] | 1.02 [1.01; 1.03] | **3.82 [1.84; 5.79]** | 1.64 [1.52; 1.77] | 1.01 [1.00; 1.02] | **2.58 [1.05; 4.12]** | 2.17 [1.78; 2.65] | 1.04 [1.01; 1.06] | **6.65 [2.55; 10.75]** |
|  | **Anxiety** | 2.18 [2.09; 2.28] | 1.00 [1.00; 1.01] | **0.68 [0.09; 1.27]** | 2.34 [2.17; 2.52] | 1.00 [1.00; 1.00] | **-0.04 [-0.47; 0.39]** | 2.11 [2.01; 2.23] | 1.01 [1.00; 1.01] | **1.00 [0.26; 1.73]** | 2.04 [1.94; 2.16] | 1.00 [1.00; 1.01] | **0.43 [-0.21; 1.07]** | 2.43 [2.25; 2.62] | 1.01 [1.00; 1.02] | **1.29 [0.01; 2.58]** |
|  | **Depression** | 2.26 [2.19; 2.33] | 1.01 [1.00; 1.01] | **1.36 [0.83; 1.89]** | 2.31 [2.19; 2.44] | 1.00 [1.00; 1.00] | **0.20 [-0.15; 0.56]** | 2.23 [2.14; 2.31] | 1.01 [1.01; 1.01] | **1.81 [1.12; 2.49]** | 2.11 [2.03; 2.19] | 1.01 [1.00; 1.01] | **0.97 [0.40; 1.54]** | 2.55 [2.41; 2.71] | 1.01 [1.01; 1.02] | **2.25 [1.13; 3.38]** |
| **Neglect** | **Any cancer** | 1.08 [1.01; 1.16] | 1.00 [1.00; 1.00] | **1.08 [1.01; 1.16]** | 1.05 [0.94; 1.16] | 1.00 [1.00; 1.00] | **1.05 [0.94; 1.16]** | 1.10 [1.01; 1.20] | 1.00 [1.00; 1.00] | **1.10 [1.01; 1.20]** | 1.06 [0.99; 1.14] | 1.00 [1.00; 1.00] | **1.06 [0.99; 1.14]** | 1.51 [1.15; 1.98] | 1.00 [0.99; 1.01] | **1.51 [1.15; 1.98]** |
|  | **Smoking-rel. cancer** | 1.12 [1.02; 1.24] | 1.00 [1.00; 1.00] | **1.12 [1.02; 1.24]** | 1.13 [0.93; 1.38] | 1.00 [1.00; 1.00] | **1.13 [0.93; 1.38]** | 1.11 [1.00; 1.24] | 1.00 [1.00; 1.00] | **1.11 [1.00; 1.24]** | 1.07 [0.97; 1.19] | 1.00 [1.00; 1.00] | **1.07 [0.97; 1.19]** | 2.00 [1.39; 2.87] | 1.00 [0.99; 1.02] | **2.00 [1.39; 2.87]** |
|  | **Obesity-rel. cancer** | 1.02 [0.94; 1.10] | 1.00 [1.00; 1.00] | **1.02 [0.94; 1.10]** | 1.00 [0.88; 1.13] | 1.00 [1.00; 1.00] | **1.00 [0.88; 1.13]** | 1.01 [0.92; 1.12] | 1.00 [1.00; 1.00] | **1.01 [0.92; 1.12]** | 1.01 [0.93; 1.09] | 1.00 [1.00; 1.00] | **1.01 [0.93; 1.09]** | 1.16 [0.78; 1.72] | 1.00 [0.99; 1.01] | **1.16 [0.78; 1.72]** |
|  | **MI** | 1.00 [0.90; 1.12] | 1.00 [1.00; 1.00] | **1.00 [0.90; 1.12]** | 0.92 [0.81; 1.04] | 1.00 [1.00; 1.00] | **0.92 [0.81; 1.04]** | 1.48 [1.15; 1.89] | 1.01 [1.00; 1.02] | **1.48 [1.15; 1.89]** | 0.99 [0.89; 1.11] | 1.00 [1.00; 1.00] | **0.99 [0.89; 1.11]** | 1.36 [0.77; 2.40] | 1.00 [0.99; 1.01] | **1.36 [0.77; 2.40]** |
|  | **Stroke** | 1.30 [1.17; 1.46] | 1.00 [1.00; 1.00] | **1.30 [1.17; 1.46]** | 1.27 [1.11; 1.46] | 1.00 [1.00; 1.00] | **1.27 [1.11; 1.46]** | 1.35 [1.13; 1.61] | 1.00 [1.00; 1.01] | **1.35 [1.13; 1.61]** | 1.26 [1.12; 1.41] | 1.00 [1.00; 1.00] | **1.26 [1.12; 1.41]** | 2.10 [1.44; 3.07] | 1.00 [0.99; 1.01] | **2.10 [1.44; 3.07]** |
|  | **Diabetes** | 1.08 [1.01; 1.15] | 1.00 [1.00; 1.01] | **1.08 [1.01; 1.15]** | 1.04 [0.96; 1.13] | 1.00 [0.99; 1.00] | **1.04 [0.96; 1.13]** | 1.13 [1.01; 1.25] | 1.02 [1.00; 1.03] | **1.13 [1.01; 1.25]** | 1.06 [1.00; 1.14] | 1.00 [1.00; 1.01] | **1.06 [1.00; 1.14]** | 1.34 [1.00; 1.79] | 1.02 [0.98; 1.06] | **1.34 [1.00; 1.79]** |
|  | **COPD** | 1.41 [1.31; 1.52] | 1.00 [1.00; 1.01] | **1.41 [1.31; 1.52]** | 1.22 [1.09; 1.36] | 1.00 [1.00; 1.00] | **1.22 [1.09; 1.36]** | 1.57 [1.43; 1.73] | 1.01 [1.00; 1.01] | **1.57 [1.43; 1.73]** | 1.36 [1.26; 1.47] | 1.00 [1.00; 1.01] | **1.36 [1.26; 1.47]** | 1.87 [1.48; 2.35] | 1.01 [0.99; 1.02] | **1.87 [1.48; 2.35]** |
|  | **Anxiety** | 1.72 [1.64; 1.80] | 1.00 [1.00; 1.00] | **1.72 [1.64; 1.80]** | 1.69 [1.56; 1.83] | 1.00 [1.00; 1.00] | **1.69 [1.56; 1.83]** | 1.73 [1.64; 1.84] | 1.00 [1.00; 1.01] | **1.73 [1.64; 1.84]** | 1.69 [1.60; 1.78] | 1.00 [1.00; 1.00] | **1.69 [1.60; 1.78]** | 1.80 [1.64; 1.98] | 1.00 [1.00; 1.00] | **1.80 [1.64; 1.98]** |
|  | **Depression** | 1.75 [1.69; 1.81] | 1.00 [1.00; 1.00] | **1.75 [1.69; 1.81]** | 1.66 [1.57; 1.76] | 1.00 [1.00; 1.00] | **1.66 [1.57; 1.76]** | 1.80 [1.72; 1.87] | 1.01 [1.00; 1.01] | **1.80 [1.72; 1.87]** | 1.68 [1.62; 1.75] | 1.00 [1.00; 1.00] | **1.68 [1.62; 1.75]** | 1.97 [1.84; 2.11] | 1.00 [1.00; 1.01] | **1.97 [1.84; 2.11]** |

*The analyses compared participants who were overweight during adulthood (18+) before diagnosis to those who were not, excluding participants who were solely overweight before diagnosis due to a higher weight compared to their peers at age 10. Regression and mediation models were adjusted for age at NAKO baseline examination, study center, and educational years. Sex was used as a covariate for the whole sample and the birth cohort subgroups. Smoking status was defined before disease diagnosis. PDE=pure direct effect; TIE=total indirect effect; rel.=related; MI=myocardial infarction; diabetes=type 2 diabetes; COPD=chronic bronchitis or chronic obstructive pulmonary disease; Anxiety=anxiety disorder or panic attacks; RR=risk ratio; 95%-CI=95% confidence interval; %=proportion mediated*

**Table S8. Four-way decomposition** of the mediation analyses between childhood maltreatment and the presence of the diseases via **ever smoking**.

|  |  | **Whole sample** | | | | **Men** | | | | **Women** | | | | **Older cohort (born <=1970)** | | | | **Younger cohort (born >1970)** | | | |
| --- | --- | --- | --- | --- | --- | --- | --- | --- | --- | --- | --- | --- | --- | --- | --- | --- | --- | --- | --- | --- | --- |
|  |  | **CDE** | **PIE** | **INT_re_** | **INT_med_** | **CDE** | **PIE** | **INT_re_** | **INT_med_** | **CDE** | **PIE** | **INT_re_** | **INT_med_** | **CDE** | **PIE** | **INT_re_** | **INT_med_** | **CDE** | **PIE** | **INT_re_** | **INT_med_** |
|  |  | **RR [95%-CI]** | **RR [95%-CI]** | **% [95%-CI]** | **RR [95%-CI]** | **RR [95%-CI]** | **RR [95%-CI]** | **% [95%-CI]** | **RR [95%-CI]** | **RR [95%-CI]** | **RR [95%-CI]** | **% [95%-CI]** | **RR [95%-CI]** | **RR [95%-CI]** | **RR [95%-CI]** | **% [95%-CI]** | **RR [95%-CI]** | **RR [95%-CI]** | **RR [95%-CI]** | **% [95%-CI]** | **RR [95%-CI]** |
| **Abuse** | **Any cancer** | 1.11 [0.99; 1.24] | 1.01 [1.01; 1.02] | 1.03 [0.90; 1.17] | 1.01 [0.99; 1.02] | 0.98 [0.79; 1.22] | 1.00 [0.99; 1.01] | 1.05 [0.82; 1.34] | 1.01 [0.98; 1.04] | 1.16 [1.02; 1.32] | 1.02 [1.01; 1.03] | 1.02 [0.87; 1.19] | 1.01 [0.98; 1.03] | 1.09 [0.97; 1.22] | 1.01 [1.00; 1.02] | 1.02 [0.89; 1.16] | 1.00 [0.99; 1.02] | 2.18 [2.06; 2.32] | 1.02 [1.02; 1.03] | 0.95 [0.89; 1.02] | 0.99 [0.98; 1.00] |
|  | **Smoking-rel. cancer** | 1.17 [1.00; 1.37] | 1.03 [1.02; 1.04] | 1.02 [0.85; 1.23] | 1.00 [0.98; 1.03] | 0.67 [0.37; 1.21] | 1.05 [1.03; 1.07] | 1.45 [0.77; 2.73] | 1.05 [1.00; 1.11] | 1.21 [1.03; 1.43] | 1.03 [1.01; 1.04] | 1.01 [0.83; 1.23] | 1.00 [0.98; 1.03] | 1.13 [0.96; 1.33] | 1.03 [1.02; 1.04] | 1.02 [0.84; 1.23] | 1.00 [0.98; 1.03] | 1.24 [0.85; 1.81] | 1.01 [0.99; 1.04] | 1.19 [0.77; 1.85] | 1.04 [0.98; 1.10] |
|  | **Obesity-rel. cancer** | 1.09 [0.97; 1.24] | 1.01 [1.00; 1.02] | 0.99 [0.85; 1.14] | 1.00 [0.98; 1.02] | 0.99 [0.78; 1.27] | 1.00 [0.99; 1.01] | 1.03 [0.77; 1.37] | 1.01 [0.97; 1.04] | 1.13 [0.97; 1.30] | 1.01 [1.00; 1.02] | 0.98 [0.82; 1.17] | 0.99 [0.97; 1.02] | 1.09 [0.96; 1.25] | 1.01 [1.00; 1.02] | 0.97 [0.83; 1.13] | 0.99 [0.97; 1.01] | 1.48 [0.82; 2.66] | 1.07 [1.03; 1.11] | 1.06 [0.54; 2.08] | 1.01 [0.94; 1.09] |
|  | **MI** | 1.10 [0.85; 1.43] | 1.08 [1.07; 1.09] | 1.07 [0.80; 1.42] | 1.01 [0.98; 1.04] | 0.97 [0.70; 1.35] | 1.08 [1.06; 1.09] | 1.14 [0.80; 1.62] | 1.02 [0.98; 1.05] | 1.41 [0.91; 2.20] | 1.08 [1.04; 1.11] | 1.01 [0.61; 1.69] | 1.00 [0.94; 1.07] | 1.11 [0.85; 1.45] | 1.07 [1.06; 1.09] | 1.05 [0.79; 1.41] | 1.01 [0.98; 1.04] | 0.98 [0.59; 1.63] | 0.98 [0.95; 1.02] | 1.28 [0.70; 2.34] | 1.06 [0.98; 1.14] |
|  | **Stroke** | 1.39 [1.14; 1.70] | 1.02 [1.01; 1.04] | 1.03 [0.82; 1.29] | 1.01 [0.98; 1.03] | 1.38 [1.04; 1.83] | 1.02 [1.00; 1.04] | 0.98 [0.71; 1.35] | 1.00 [0.96; 1.03] | 1.42 [1.08; 1.87] | 1.02 [1.00; 1.05] | 1.08 [0.78; 1.49] | 1.02 [0.98; 1.07] | 1.46 [1.19; 1.79] | 1.02 [1.01; 1.04] | 0.98 [0.77; 1.24] | 1.00 [0.97; 1.02] | 0.82 [0.19; 3.50] | 1.10 [1.04; 1.16] | 1.41 [0.30; 6.75] | 1.04 [0.92; 1.18] |
|  | **Diabetes** | 1.34 [1.20; 1.50] | 1.03 [1.02; 1.04] | 0.97 [0.85; 1.11] | 0.99 [0.98; 1.01] | 1.39 [1.18; 1.63] | 1.03 [1.02; 1.04] | 0.95 [0.79; 1.14] | 0.99 [0.97; 1.01] | 1.28 [1.09; 1.51] | 1.02 [1.01; 1.03] | 1.00 [0.83; 1.22] | 1.00 [0.98; 1.03] | 1.30 [1.15; 1.46] | 1.03 [1.02; 1.03] | 0.98 [0.85; 1.12] | 1.00 [0.98; 1.01] | 0.84 [0.40; 1.75] | 1.00 [0.96; 1.05] | 1.75 [0.77; 3.96] | 1.11 [1.02; 1.20] |
|  | **COPD** | 1.60 [1.40; 1.84] | 1.07 [1.06; 1.08] | 1.01 [0.86; 1.18] | 1.00 [0.98; 1.02] | 1.53 [1.19; 1.96] | 1.07 [1.06; 1.09] | 1.01 [0.77; 1.33] | 1.00 [0.97; 1.03] | 1.61 [1.37; 1.90] | 1.07 [1.05; 1.08] | 1.03 [0.85; 1.25] | 1.01 [0.98; 1.03] | 1.60 [1.38; 1.85] | 1.07 [1.06; 1.08] | 0.98 [0.83; 1.16] | 1.00 [0.98; 1.02] | 1.95 [1.31; 2.89] | 1.02 [0.99; 1.05] | 0.95 [0.60; 1.52] | 0.99 [0.93; 1.05] |
|  | **Anxiety** | 2.28 [2.12; 2.45] | 1.05 [1.04; 1.05] | 0.93 [0.85; 1.01] | 0.99 [0.98; 1.00] | 2.42 [2.11; 2.78] | 1.04 [1.03; 1.05] | 0.95 [0.81; 1.11] | 0.99 [0.97; 1.01] | 2.23 [2.05; 2.42] | 1.05 [1.04; 1.06] | 0.93 [0.84; 1.02] | 0.98 [0.97; 0.99] | 2.01 [1.84; 2.20] | 1.03 [1.03; 1.04] | 0.99 [0.89; 1.10] | 1.00 [0.99; 1.01] | 1.56 [1.08; 2.26] | 1.05 [1.03; 1.08] | 1.20 [0.79; 1.82] | 1.03 [0.99; 1.08] |
|  | **Depression** | 2.39 [2.28; 2.51] | 1.03 [1.03; 1.03] | 0.93 [0.88; 0.98] | 0.99 [0.98; 0.99] | 2.46 [2.24; 2.70] | 1.03 [1.02; 1.04] | 0.94 [0.84; 1.04] | 0.99 [0.98; 1.00] | 2.37 [2.24; 2.51] | 1.03 [1.03; 1.04] | 0.93 [0.87; 1.00] | 0.98 [0.97; 0.99] | 1.09 [0.97; 1.22] | 1.01 [1.00; 1.02] | 1.02 [0.89; 1.16] | 1.00 [0.99; 1.02] | 2.78 [2.47; 3.13] | 1.06 [1.05; 1.07] | 0.84 [0.73; 0.97] | 0.97 [0.95; 0.99] |
| **Neglect** | **Any cancer** | 1.09 [0.99; 1.21] | 1.01 [1.00; 1.01] | 0.99 [0.87; 1.11] | 1.00 [0.99; 1.01] | 1.01 [0.85; 1.20] | 1.00 [1.00; 1.00] | 1.03 [0.84; 1.26] | 1.00 [0.99; 1.01] | 1.14 [1.01; 1.30] | 1.01 [1.01; 1.02] | 0.97 [0.83; 1.12] | 0.99 [0.98; 1.01] | 1.09 [0.98; 1.21] | 1.01 [1.00; 1.01] | 0.97 [0.86; 1.10] | 1.00 [0.99; 1.00] | 1.12 [0.70; 1.78] | 1.01 [1.00; 1.03] | 1.22 [0.71; 2.08] | 1.02 [0.99; 1.07] |
|  | **Smoking-rel. cancer** | 1.14 [0.98; 1.32] | 1.02 [1.01; 1.02] | 0.97 [0.81; 1.15] | 1.00 [0.99; 1.01] | 1.40 [0.96; 2.02] | 1.02 [1.01; 1.03] | 0.82 [0.53; 1.24] | 0.99 [0.97; 1.01] | 1.10 [0.93; 1.30] | 1.02 [1.01; 1.03] | 1.00 [0.82; 1.21] | 1.00 [0.98; 1.02] | 1.12 [0.96; 1.31] | 1.01 [1.01; 1.02] | 0.95 [0.79; 1.13] | 1.00 [0.98; 1.01] | 1.22 [0.58; 2.55] | 1.04 [1.01; 1.06] | 1.44 [0.63; 3.27] | 1.03 [0.98; 1.08] |
|  | **Obesity-rel. cancer** | 1.02 [0.91; 1.15] | 1.00 [1.00; 1.01] | 0.99 [0.86; 1.14] | 1.00 [0.99; 1.01] | 0.89 [0.72; 1.10] | 1.00 [1.00; 1.00] | 1.09 [0.85; 1.38] | 1.01 [1.00; 1.02] | 1.10 [0.95; 1.27] | 1.01 [1.00; 1.01] | 0.94 [0.79; 1.13] | 0.99 [0.97; 1.00] | 1.03 [0.91; 1.16] | 1.00 [1.00; 1.01] | 0.97 [0.84; 1.13] | 1.00 [0.99; 1.01] | 0.84 [0.44; 1.61] | 0.99 [0.97; 1.01] | 1.27 [0.60; 2.70] | 1.03 [0.98; 1.09] |
|  | **MI** | 1.07 [0.85; 1.33] | 1.04 [1.03; 1.05] | 0.94 [0.73; 1.21] | 1.00 [0.98; 1.01] | 0.99 [0.76; 1.29] | 1.03 [1.02; 1.03] | 0.93 [0.70; 1.25] | 1.00 [0.98; 1.01] | 1.31 [0.85; 2.00] | 1.04 [1.02; 1.07] | 1.09 [0.67; 1.78] | 1.01 [0.97; 1.05] | 1.05 [0.84; 1.32] | 1.03 [1.03; 1.04] | 0.94 [0.73; 1.22] | 1.00 [0.98; 1.01] | 1.36 [0.40; 4.61] | 1.06 [1.03; 1.10] | 0.96 [0.25; 3.69] | 1.00 [0.92; 1.08] |
|  | **Stroke** | 1.40 [1.17; 1.67] | 1.01 [1.01; 1.02] | 0.96 [0.78; 1.18] | 1.00 [0.98; 1.01] | 1.24 [0.97; 1.59] | 1.01 [1.00; 1.01] | 1.03 [0.77; 1.36] | 1.00 [0.99; 1.01] | 1.61 [1.24; 2.08] | 1.02 [1.01; 1.04] | 0.91 [0.66; 1.24] | 0.98 [0.96; 1.01] | 1.37 [1.14; 1.65] | 1.01 [1.01; 1.02] | 0.94 [0.76; 1.17] | 0.99 [0.98; 1.01] | 1.74 [0.94; 3.24] | 1.01 [0.99; 1.04] | 1.20 [0.59; 2.45] | 1.02 [0.97; 1.08] |
|  | **Diabetes** | 1.18 [1.06; 1.30] | 1.02 [1.01; 1.02] | 0.93 [0.82; 1.05] | 0.99 [0.98; 1.00] | 1.23 [1.07; 1.42] | 1.01 [1.01; 1.02] | 0.87 [0.74; 1.02] | 0.99 [0.98; 1.00] | 1.11 [0.95; 1.29] | 1.01 [1.01; 1.02] | 1.02 [0.85; 1.23] | 1.00 [0.99; 1.02] | 1.16 [1.04; 1.29] | 1.01 [1.01; 1.02] | 0.92 [0.81; 1.04] | 0.99 [0.99; 1.00] | 1.54 [0.99; 2.41] | 1.01 [1.00; 1.03] | 0.94 [0.56; 1.60] | 0.99 [0.95; 1.03] |
|  | **COPD** | 1.43 [1.25; 1.63] | 1.04 [1.03; 1.04] | 0.96 [0.82; 1.11] | 1.00 [0.99; 1.01] | 1.26 [1.00; 1.58] | 1.02 [1.02; 1.03] | 0.96 [0.74; 1.24] | 1.00 [0.99; 1.01] | 1.53 [1.29; 1.80] | 1.04 [1.03; 1.05] | 0.99 [0.82; 1.20] | 1.00 [0.98; 1.02] | 1.43 [1.24; 1.65] | 1.03 [1.03; 1.04] | 0.93 [0.80; 1.10] | 1.00 [0.99; 1.01] | 1.44 [0.94; 2.19] | 1.04 [1.02; 1.05] | 1.14 [0.71; 1.83] | 1.01 [0.98; 1.05] |
|  | **Anxiety** | 1.85 [1.71; 1.99] | 1.02 [1.02; 1.03] | 0.92 [0.84; 1.01] | 0.99 [0.99; 1.00] | 1.68 [1.45; 1.93] | 1.01 [1.01; 1.02] | 1.00 [0.85; 1.18] | 1.00 [0.99; 1.01] | 1.92 [1.75; 2.10] | 1.03 [1.03; 1.04] | 0.91 [0.81; 1.01] | 0.99 [0.98; 1.00] | 1.73 [1.59; 1.90] | 1.02 [1.01; 1.02] | 0.96 [0.86; 1.06] | 1.00 [0.99; 1.00] | 2.08 [1.80; 2.41] | 1.04 [1.03; 1.05] | 0.86 [0.73; 1.03] | 0.98 [0.97; 1.00] |
|  | **Depression** | 1.86 [1.76; 1.96] | 1.02 [1.01; 1.02] | 0.93 [0.88; 0.99] | 0.99 [0.99; 1.00] | 1.71 [1.56; 1.89] | 1.01 [1.01; 1.01] | 0.96 [0.86; 1.08] | 1.00 [0.99; 1.00] | 1.93 [1.81; 2.05] | 1.02 [1.02; 1.03] | 0.94 [0.87; 1.01] | 0.99 [0.98; 1.00] | 1.73 [1.63; 1.84] | 1.01 [1.01; 1.01] | 0.97 [0.90; 1.04] | 1.00 [0.99; 1.00] | 2.20 [1.99; 2.45] | 1.02 [1.02; 1.03] | 0.88 [0.78; 1.00] | 0.99 [0.98; 1.00] |

*The analyses compared never to ever smokers prior to the diagnosis. Regression and mediation models were adjusted for age at NAKO baseline examination, study center, and educational years. Sex was used as a covariate for the whole sample and the birth cohort subgroups. Smoking status was defined before disease diagnosis. CDE=controlled direct effect; PIE=pure natural indirect effect; INT_ref_=reference interaction; INT_med_=mediated interaction; rel.=related; MI=myocardial infarction; diabetes=type 2 diabetes; COPD=chronic bronchitis or chronic obstructive pulmonary disease; Anxiety=anxiety disorder or panic attacks; RR=risk ratio; 95%-CI=95% confidence interval*

**Table S9. Four-way decomposition** of the mediation analyses between childhood maltreatment and the presence of the diseases via **current smoking**.

|  |  | **Whole sample** | | | | **Men** | | | | **Women** | | | | **Older cohort (born <=1970)** | | | | **Younger cohort (born >1970)** | | | |
| --- | --- | --- | --- | --- | --- | --- | --- | --- | --- | --- | --- | --- | --- | --- | --- | --- | --- | --- | --- | --- | --- |
|  |  | **CDE** | **PIE** | **INT_re_** | **INT_med_** | **CDE** | **PIE** | **INT_re_** | **INT_med_** | **CDE** | **PIE** | **INT_re_** | **INT_med_** | **CDE** | **PIE** | **INT_re_** | **INT_med_** | **CDE** | **PIE** | **INT_re_** | **INT_med_** |
|  |  | **RR [95%-CI]** | **RR [95%-CI]** | **% [95%-CI]** | **RR [95%-CI]** | **RR [95%-CI]** | **RR [95%-CI]** | **% [95%-CI]** | **RR [95%-CI]** | **RR [95%-CI]** | **RR [95%-CI]** | **% [95%-CI]** | **RR [95%-CI]** | **RR [95%-CI]** | **RR [95%-CI]** | **% [95%-CI]** | **RR [95%-CI]** | **RR [95%-CI]** | **RR [95%-CI]** | **% [95%-CI]** | **RR [95%-CI]** |
| **Abuse** | **Any cancer** | 1.10 [0.99; 1.23] | 1.08 [1.06; 1.09] | 1.02 [0.89; 1.17] | 1.01 [0.98; 1.03] | 0.98 [0.79; 1.22] | 1.05 [1.03; 1.07] | 1.05 [0.81; 1.37] | 1.02 [0.98; 1.06] | 1.16 [1.02; 1.32] | 1.08 [1.07; 1.10] | 0.99 [0.85; 1.17] | 1.00 [0.97; 1.03] | 1.08 [0.97; 1.22] | 1.07 [1.06; 1.08] | 1.00 [0.87; 1.16] | 1.00 [0.98; 1.03] | 1.23 [0.84; 1.80] | 1.08 [1.04; 1.12] | 1.17 [0.74; 1.83] | 1.04 [0.97; 1.12] |
|  | **Smoking-rel. cancer** | 1.16 [0.99; 1.36] | 1.12 [1.10; 1.14] | 0.98 [0.81; 1.19] | 1.00 [0.96; 1.03] | 0.66 [0.37; 1.20] | 1.14 [1.11; 1.18] | 1.28 [0.66; 2.49] | 1.06 [0.97; 1.15] | 1.22 [1.03; 1.43] | 1.10 [1.08; 1.12] | 0.98 [0.80; 1.21] | 0.99 [0.96; 1.03] | 1.13 [0.96; 1.33] | 1.11 [1.09; 1.13] | 0.97 [0.79; 1.19] | 0.99 [0.95; 1.03] | 1.49 [0.83; 2.67] | 1.16 [1.11; 1.22] | 1.05 [0.53; 2.09] | 1.01 [0.92; 1.11] |
|  | **Obesity-rel. cancer** | 1.09 [0.96; 1.24] | 1.05 [1.04; 1.07] | 0.99 [0.84; 1.16] | 0.99 [0.97; 1.02] | 0.99 [0.77; 1.27] | 1.04 [1.02; 1.06] | 1.05 [0.78; 1.43] | 1.02 [0.97; 1.07] | 1.13 [0.97; 1.31] | 1.06 [1.04; 1.07] | 0.96 [0.80; 1.16] | 0.98 [0.95; 1.01] | 1.09 [0.96; 1.24] | 1.05 [1.04; 1.07] | 0.97 [0.82; 1.15] | 0.99 [0.96; 1.02] | 0.97 [0.58; 1.61] | 1.02 [0.97; 1.07] | 1.18 [0.63; 2.21] | 1.06 [0.96; 1.18] |
|  | **MI** | 1.11 [0.85; 1.43] | 1.24 [1.21; 1.26] | 1.06 [0.79; 1.42] | 1.01 [0.97; 1.05] | 0.97 [0.70; 1.34] | 1.22 [1.19; 1.26] | 1.12 [0.78; 1.60] | 1.02 [0.97; 1.07] | 1.42 [0.91; 2.21] | 1.20 [1.14; 1.26] | 1.07 [0.63; 1.81] | 1.02 [0.93; 1.12] | 1.12 [0.86; 1.45] | 1.23 [1.21; 1.26] | 1.04 [0.77; 1.40] | 1.01 [0.96; 1.06] | 0.83 [0.19; 3.57] | 1.21 [1.14; 1.29] | 1.37 [0.28; 6.63] | 1.04 [0.90; 1.21] |
|  | **Stroke** | 1.39 [1.14; 1.69] | 1.12 [1.09; 1.14] | 0.99 [0.78; 1.26] | 1.00 [0.96; 1.04] | 1.37 [1.03; 1.82] | 1.11 [1.09; 1.14] | 0.91 [0.64; 1.28] | 0.97 [0.92; 1.02] | 1.41 [1.07; 1.86] | 1.10 [1.06; 1.14] | 1.08 [0.77; 1.52] | 1.04 [0.97; 1.10] | 1.46 [1.19; 1.79] | 1.12 [1.09; 1.14] | 0.96 [0.75; 1.23] | 0.99 [0.94; 1.03] | 0.85 [0.41; 1.77] | 1.09 [1.03; 1.15] | 1.43 [0.62; 3.32] | 1.09 [0.97; 1.22] |
|  | **Diabetes** | 1.33 [1.19; 1.49] | 1.08 [1.07; 1.10] | 0.97 [0.85; 1.12] | 0.99 [0.97; 1.02] | 1.37 [1.17; 1.61] | 1.09 [1.07; 1.10] | 0.93 [0.76; 1.13] | 0.98 [0.94; 1.01] | 1.28 [1.09; 1.50] | 1.05 [1.03; 1.07] | 1.04 [0.85; 1.27] | 1.02 [0.98; 1.06] | 1.29 [1.14; 1.45] | 1.08 [1.06; 1.09] | 0.97 [0.84; 1.13] | 0.99 [0.96; 1.02] | 1.96 [1.32; 2.90] | 1.08 [1.04; 1.13] | 0.92 [0.57; 1.48] | 0.97 [0.90; 1.05] |
|  | **COPD** | 1.60 [1.39; 1.83] | 1.22 [1.20; 1.24] | 0.98 [0.84; 1.15] | 1.00 [0.97; 1.02] | 1.51 [1.17; 1.94] | 1.22 [1.19; 1.25] | 1.00 [0.75; 1.32] | 1.00 [0.96; 1.04] | 1.60 [1.36; 1.89] | 1.20 [1.17; 1.23] | 1.01 [0.83; 1.22] | 1.00 [0.96; 1.04] | 1.60 [1.38; 1.86] | 1.22 [1.20; 1.25] | 0.96 [0.81; 1.14] | 0.99 [0.96; 1.02] | 1.55 [1.07; 2.24] | 1.16 [1.12; 1.20] | 1.12 [0.74; 1.71] | 1.02 [0.97; 1.09] |
|  | **Anxiety** | 2.26 [2.10; 2.42] | 1.14 [1.13; 1.16] | 0.93 [0.85; 1.01] | 0.98 [0.96; 0.99] | 2.37 [2.07; 2.73] | 1.14 [1.12; 1.16] | 0.94 [0.79; 1.10] | 0.98 [0.95; 1.01] | 2.21 [2.04; 2.41] | 1.14 [1.12; 1.16] | 0.94 [0.85; 1.04] | 0.97 [0.95; 0.99] | 2.01 [1.84; 2.20] | 1.13 [1.12; 1.15] | 0.98 [0.88; 1.09] | 0.99 [0.97; 1.01] | 2.77 [2.46; 3.12] | 1.15 [1.13; 1.17] | 0.83 [0.72; 0.96] | 0.95 [0.92; 0.98] |
|  | **Depression** | 2.38 [2.27; 2.50] | 1.12 [1.11; 1.13] | 0.91 [0.85; 0.96] | 0.96 [0.95; 0.98] | 2.43 [2.21; 2.67] | 1.12 [1.10; 1.13] | 0.92 [0.82; 1.03] | 0.97 [0.95; 0.99] | 2.36 [2.23; 2.50] | 1.12 [1.10; 1.13] | 0.92 [0.86; 0.99] | 0.96 [0.95; 0.98] | 2.19 [2.07; 2.33] | 1.12 [1.10; 1.13] | 0.93 [0.86; 1.00] | 0.97 [0.95; 0.99] | 2.80 [2.57; 3.05] | 1.12 [1.10; 1.14] | 0.86 [0.78; 0.96] | 0.96 [0.94; 0.98] |
| **Neglect** | **Any cancer** | 1.09 [0.98; 1.21] | 1.05 [1.04; 1.06] | 0.99 [0.87; 1.13] | 1.00 [0.98; 1.01] | 1.01 [0.85; 1.21] | 1.02 [1.01; 1.03] | 1.08 [0.87; 1.34] | 1.01 [0.99; 1.03] | 1.13 [1.00; 1.28] | 1.07 [1.06; 1.09] | 0.95 [0.81; 1.11] | 0.98 [0.96; 1.01] | 1.09 [0.98; 1.21] | 1.05 [1.04; 1.06] | 0.98 [0.86; 1.12] | 0.99 [0.98; 1.01] | 1.10 [0.69; 1.75] | 1.06 [1.03; 1.08] | 1.17 [0.68; 2.03] | 1.03 [0.97; 1.09] |
|  | **Smoking-rel. cancer** | 1.12 [0.97; 1.31] | 1.08 [1.07; 1.09] | 0.96 [0.79; 1.15] | 0.99 [0.97; 1.02] | 1.36 [0.94; 1.98] | 1.07 [1.05; 1.10] | 0.90 [0.58; 1.40] | 0.99 [0.95; 1.02] | 1.08 [0.92; 1.28] | 1.09 [1.07; 1.10] | 0.97 [0.79; 1.19] | 0.99 [0.96; 1.02] | 1.11 [0.95; 1.30] | 1.07 [1.06; 1.09] | 0.94 [0.77; 1.14] | 0.98 [0.96; 1.01] | 1.21 [0.58; 2.54] | 1.10 [1.06; 1.15] | 1.35 [0.59; 3.10] | 1.04 [0.96; 1.12] |
|  | **Obesity-rel. cancer** | 1.02 [0.90; 1.15] | 1.04 [1.03; 1.05] | 0.98 [0.84; 1.14] | 0.99 [0.98; 1.01] | 0.89 [0.72; 1.10] | 1.02 [1.01; 1.03] | 1.12 [0.86; 1.45] | 1.02 [1.00; 1.04] | 1.09 [0.94; 1.26] | 1.05 [1.03; 1.06] | 0.92 [0.77; 1.12] | 0.97 [0.94; 1.00] | 1.03 [0.91; 1.16] | 1.03 [1.02; 1.04] | 0.97 [0.83; 1.13] | 0.99 [0.97; 1.01] | 0.82 [0.43; 1.56] | 1.02 [0.99; 1.05] | 1.12 [0.51; 2.47] | 1.03 [0.94; 1.12] |
|  | **MI** | 1.05 [0.84; 1.31] | 1.15 [1.14; 1.17] | 0.88 [0.68; 1.14] | 0.98 [0.96; 1.01] | 0.97 [0.74; 1.26] | 1.10 [1.08; 1.13] | 0.86 [0.64; 1.17] | 0.99 [0.96; 1.02] | 1.30 [0.85; 1.98] | 1.16 [1.12; 1.21] | 1.06 [0.64; 1.77] | 1.02 [0.94; 1.09] | 1.04 [0.83; 1.31] | 1.15 [1.13; 1.18] | 0.88 [0.68; 1.14] | 0.98 [0.95; 1.01] | 1.41 [0.41; 4.77] | 1.15 [1.10; 1.21] | 0.96 [0.25; 3.76] | 1.00 [0.89; 1.12] |
|  | **Stroke** | 1.38 [1.16; 1.65] | 1.08 [1.06; 1.09] | 0.96 [0.77; 1.20] | 0.99 [0.97; 1.02] | 1.22 [0.95; 1.56] | 1.05 [1.03; 1.07] | 1.01 [0.75; 1.36] | 1.00 [0.97; 1.03] | 1.60 [1.23; 2.07] | 1.10 [1.07; 1.13] | 0.93 [0.67; 1.29] | 0.98 [0.93; 1.03] | 1.36 [1.13; 1.64] | 1.08 [1.06; 1.09] | 0.95 [0.75; 1.19] | 0.99 [0.96; 1.02] | 1.81 [0.97; 3.36] | 1.07 [1.03; 1.11] | 1.05 [0.50; 2.19] | 1.01 [0.93; 1.10] |
|  | **Diabetes** | 1.17 [1.05; 1.29] | 1.06 [1.05; 1.07] | 0.94 [0.82; 1.07] | 0.98 [0.97; 1.00] | 1.22 [1.06; 1.40] | 1.04 [1.03; 1.05] | 0.89 [0.75; 1.05] | 0.98 [0.97; 1.00] | 1.10 [0.94; 1.29] | 1.05 [1.03; 1.07] | 1.02 [0.83; 1.24] | 1.01 [0.98; 1.04] | 1.15 [1.04; 1.28] | 1.05 [1.04; 1.06] | 0.94 [0.82; 1.07] | 0.98 [0.97; 1.00] | 1.57 [1.00; 2.46] | 1.06 [1.03; 1.09] | 0.90 [0.52; 1.55] | 0.98 [0.92; 1.04] |
|  | **COPD** | 1.40 [1.23; 1.60] | 1.14 [1.12; 1.16] | 0.93 [0.79; 1.08] | 0.99 [0.97; 1.01] | 1.22 [0.97; 1.54] | 1.10 [1.08; 1.12] | 0.93 [0.71; 1.21] | 0.99 [0.96; 1.03] | 1.51 [1.28; 1.78] | 1.17 [1.14; 1.19] | 0.96 [0.79; 1.17] | 0.99 [0.96; 1.03] | 1.42 [1.23; 1.64] | 1.14 [1.12; 1.16] | 0.89 [0.76; 1.06] | 0.98 [0.96; 1.01] | 1.44 [0.94; 2.20] | 1.11 [1.08; 1.14] | 1.11 [0.68; 1.79] | 1.01 [0.96; 1.07] |
|  | **Anxiety** | 1.79 [1.66; 1.93] | 1.10 [1.09; 1.11] | 0.91 [0.83; 1.00] | 0.98 [0.97; 1.00] | 1.60 [1.39; 1.85] | 1.07 [1.05; 1.08] | 1.00 [0.84; 1.18] | 1.00 [0.98; 1.02] | 1.88 [1.72; 2.06] | 1.12 [1.10; 1.13] | 0.89 [0.80; 1.00] | 0.96 [0.94; 0.99] | 1.71 [1.57; 1.88] | 1.09 [1.08; 1.10] | 0.94 [0.84; 1.04] | 0.99 [0.97; 1.00] | 2.08 [1.80; 2.41] | 1.10 [1.08; 1.12] | 0.82 [0.69; 0.98] | 0.96 [0.94; 0.99] |
|  | **Depression** | 1.82 [1.72; 1.92] | 1.08 [1.07; 1.09] | 0.91 [0.85; 0.97] | 0.98 [0.97; 0.99] | 1.65 [1.50; 1.82] | 1.06 [1.04; 1.07] | 0.95 [0.85; 1.07] | 0.99 [0.98; 1.01] | 1.89 [1.78; 2.02] | 1.09 [1.08; 1.11] | 0.91 [0.84; 0.98] | 0.97 [0.95; 0.98] | 1.72 [1.62; 1.83] | 1.08 [1.07; 1.09] | 0.93 [0.86; 1.00] | 0.98 [0.97; 0.99] | 2.20 [1.98; 2.44] | 1.08 [1.07; 1.10] | 0.84 [0.74; 0.95] | 0.96 [0.94; 0.98] |

*The analyses compared never to current smokers prior to the diagnosis by excluding participants who both started and quit smoking before the respective diagnosis. Regression and mediation models were adjusted for age at NAKO baseline examination, study center, and educational years. Sex was used as a covariate for the whole sample and the birth cohort subgroups. Smoking status was defined before disease diagnosis. CDE=controlled direct effect; PIE=pure natural indirect effect; INT_ref_=reference interaction; INT_med_=mediated interaction; rel.=related; MI=myocardial infarction; diabetes=type 2 diabetes; COPD=chronic bronchitis or chronic obstructive pulmonary disease; Anxiety=anxiety disorder or panic attacks; RR=risk ratio; 95%-CI=95% confidence interval*

**Table S10. Four-way decomposition** of the mediation analyses between childhood maltreatment and the presence of the diseases via **early-onset overweight**.

|  |  | **Whole sample** | | | | **Men** | | | | **Women** | | | | **Older cohort (born <=1970)** | | | | **Younger cohort (born >1970)** | | | |
| --- | --- | --- | --- | --- | --- | --- | --- | --- | --- | --- | --- | --- | --- | --- | --- | --- | --- | --- | --- | --- | --- |
|  |  | **CDE** | **PIE** | **INT_re_** | **INT_med_** | **CDE** | **PIE** | **INT_re_** | **INT_med_** | **CDE** | **PIE** | **INT_re_** | **INT_med_** | **CDE** | **PIE** | **INT_re_** | **INT_med_** | **CDE** | **PIE** | **INT_re_** | **INT_med_** |
|  |  | **RR [95%-CI]** | **RR [95%-CI]** | **% [95%-CI]** | **RR [95%-CI]** | **RR [95%-CI]** | **RR [95%-CI]** | **% [95%-CI]** | **RR [95%-CI]** | **RR [95%-CI]** | **RR [95%-CI]** | **% [95%-CI]** | **RR [95%-CI]** | **RR [95%-CI]** | **RR [95%-CI]** | **% [95%-CI]** | **RR [95%-CI]** | **RR [95%-CI]** | **RR [95%-CI]** | **% [95%-CI]** | **RR [95%-CI]** |
| **Abuse** | **Any cancer** | 1.15 [1.07; 1.24] | 1.00 [1.00; 1.01] | 1.01 [0.91; 1.11] | 1.00 [0.99; 1.01] | 1.00 [0.88; 1.13] | 1.00 [1.00; 1.00] | 1.05 [0.88; 1.25] | 1.01 [1.00; 1.01] | 1.23 [1.13; 1.34] | 1.00 [1.00; 1.01] | 0.99 [0.88; 1.11] | 1.00 [0.99; 1.01] | 1.12 [1.04; 1.21] | 1.00 [1.00; 1.01] | 1.00 [0.90; 1.10] | 1.00 [0.99; 1.01] | 1.42 [1.09; 1.83] | 1.01 [0.99; 1.02] | 1.09 [0.78; 1.54] | 1.02 [0.99; 1.05] |
|  | **Smoking-rel. cancer** | 1.27 [1.14; 1.40] | 1.01 [1.00; 1.01] | 0.97 [0.84; 1.11] | 0.99 [0.98; 1.00] | 1.06 [0.82; 1.36] | 1.01 [1.00; 1.01] | 1.03 [0.74; 1.44] | 1.00 [0.99; 1.02] | 1.30 [1.17; 1.45] | 1.00 [1.00; 1.01] | 0.97 [0.84; 1.13] | 0.99 [0.98; 1.00] | 1.22 [1.10; 1.35] | 1.01 [1.00; 1.01] | 0.96 [0.83; 1.11] | 0.99 [0.98; 1.00] | 1.63 [1.14; 2.33] | 1.01 [0.98; 1.03] | 1.03 [0.64; 1.67] | 1.01 [0.96; 1.05] |
|  | **Obesity-rel. cancer** | 1.06 [0.98; 1.16] | 1.00 [1.00; 1.01] | 1.02 [0.91; 1.14] | 1.00 [1.00; 1.01] | 0.97 [0.83; 1.12] | 1.00 [1.00; 1.00] | 1.07 [0.88; 1.31] | 1.01 [1.00; 1.02] | 1.11 [1.00; 1.23] | 1.00 [1.00; 1.01] | 0.99 [0.86; 1.15] | 1.00 [0.99; 1.01] | 1.05 [0.96; 1.15] | 1.00 [1.00; 1.00] | 1.01 [0.89; 1.14] | 1.00 [0.99; 1.01] | 1.11 [0.77; 1.60] | 1.01 [0.99; 1.03] | 1.18 [0.73; 1.91] | 1.03 [0.99; 1.08] |
|  | **MI** | 1.29 [1.13; 1.46] | 1.01 [1.01; 1.02] | 0.97 [0.82; 1.15] | 0.99 [0.98; 1.01] | 1.25 [1.08; 1.44] | 1.01 [1.00; 1.01] | 0.96 [0.79; 1.16] | 0.99 [0.99; 1.00] | 1.50 [1.13; 1.99] | 1.03 [1.01; 1.05] | 0.99 [0.68; 1.44] | 1.00 [0.96; 1.03] | 1.29 [1.13; 1.47] | 1.01 [1.01; 1.02] | 0.96 [0.81; 1.14] | 0.99 [0.98; 1.00] | 0.85 [0.38; 1.89] | 1.03 [1.00; 1.07] | 1.51 [0.57; 3.97] | 1.06 [0.99; 1.15] |
|  | **Stroke** | 1.41 [1.24; 1.59] | 1.01 [1.00; 1.01] | 1.04 [0.88; 1.23] | 1.01 [1.00; 1.02] | 1.36 [1.16; 1.61] | 1.00 [1.00; 1.01] | 1.00 [0.80; 1.24] | 1.00 [0.99; 1.01] | 1.49 [1.23; 1.79] | 1.02 [1.00; 1.03] | 1.06 [0.82; 1.36] | 1.02 [0.99; 1.04] | 1.40 [1.23; 1.59] | 1.01 [1.00; 1.01] | 1.03 [0.87; 1.23] | 1.01 [0.99; 1.02] | 1.47 [0.97; 2.22] | 1.01 [0.98; 1.03] | 1.11 [0.64; 1.90] | 1.02 [0.97; 1.07] |
|  | **Diabetes** | 1.31 [1.22; 1.42] | 1.04 [1.03; 1.05] | 0.98 [0.89; 1.09] | 1.00 [0.99; 1.01] | 1.31 [1.18; 1.44] | 1.02 [1.01; 1.03] | 1.01 [0.88; 1.14] | 1.00 [0.99; 1.01] | 1.34 [1.19; 1.51] | 1.05 [1.04; 1.07] | 0.95 [0.81; 1.11] | 0.99 [0.97; 1.00] | 1.29 [1.20; 1.40] | 1.03 [1.02; 1.04] | 0.97 [0.88; 1.08] | 1.00 [0.98; 1.01] | 1.61 [1.12; 2.32] | 1.10 [1.07; 1.13] | 1.07 [0.69; 1.65] | 1.01 [0.96; 1.05] |
|  | **COPD** | 1.67 [1.54; 1.81] | 1.02 [1.01; 1.02] | 1.02 [0.92; 1.13] | 1.00 [0.99; 1.01] | 1.55 [1.37; 1.75] | 1.01 [1.00; 1.01] | 1.05 [0.90; 1.24] | 1.01 [1.00; 1.02] | 1.77 [1.60; 1.96] | 1.03 [1.02; 1.03] | 0.99 [0.86; 1.13] | 1.00 [0.98; 1.01] | 1.62 [1.49; 1.76] | 1.01 [1.01; 1.02] | 1.02 [0.91; 1.14] | 1.00 [0.99; 1.01] | 1.96 [1.55; 2.46] | 1.03 [1.02; 1.05] | 1.01 [0.75; 1.36] | 1.00 [0.97; 1.03] |
|  | **Anxiety** | 2.22 [2.12; 2.33] | 1.02 [1.01; 1.02] | 0.97 [0.91; 1.03] | 0.99 [0.99; 1.00] | 2.41 [2.22; 2.61] | 1.01 [1.00; 1.01] | 0.97 [0.87; 1.08] | 1.00 [0.99; 1.00] | 2.15 [2.03; 2.27] | 1.02 [1.02; 1.03] | 0.97 [0.90; 1.05] | 0.99 [0.98; 1.00] | 2.10 [1.98; 2.22] | 1.01 [1.01; 1.02] | 0.97 [0.90; 1.04] | 0.99 [0.99; 1.00] | 2.45 [2.26; 2.66] | 1.02 [1.01; 1.03] | 0.97 [0.87; 1.08] | 0.99 [0.98; 1.00] |
|  | **Depression** | 2.30 [2.22; 2.38] | 1.02 [1.02; 1.02] | 0.96 [0.92; 1.01] | 0.99 [0.99; 1.00] | 2.39 [2.26; 2.54] | 1.01 [1.00; 1.01] | 0.96 [0.89; 1.04] | 1.00 [0.99; 1.00] | 2.27 [2.17; 2.36] | 1.03 [1.02; 1.03] | 0.97 [0.91; 1.02] | 0.99 [0.98; 1.00] | 2.16 [2.07; 2.24] | 1.02 [1.01; 1.02] | 0.97 [0.91; 1.02] | 0.99 [0.99; 1.00] | 2.61 [2.45; 2.78] | 1.03 [1.02; 1.03] | 0.96 [0.88; 1.04] | 0.99 [0.98; 1.00] |
| **Neglect** | **Any cancer** | 1.09 [1.01; 1.17] | 1.00 [1.00; 1.00] | 1.00 [0.90; 1.10] | 1.00 [1.00; 1.00] | 1.02 [0.91; 1.15] | 1.00 [1.00; 1.00] | 1.02 [0.88; 1.19] | 1.00 [1.00; 1.00] | 1.13 [1.03; 1.23] | 1.00 [1.00; 1.01] | 0.98 [0.87; 1.11] | 1.00 [0.99; 1.00] | 1.07 [0.99; 1.15] | 1.00 [1.00; 1.00] | 1.00 [0.90; 1.10] | 1.00 [1.00; 1.00] | 1.46 [1.09; 1.97] | 1.01 [1.00; 1.01] | 0.96 [0.64; 1.43] | 1.00 [0.98; 1.01] |
|  | **Smoking-rel. cancer** | 1.13 [1.02; 1.25] | 1.00 [1.00; 1.00] | 0.98 [0.85; 1.13] | 1.00 [0.99; 1.00] | 1.15 [0.93; 1.44] | 1.00 [1.00; 1.00] | 0.96 [0.71; 1.28] | 1.00 [1.00; 1.00] | 1.12 [1.00; 1.26] | 1.00 [1.00; 1.00] | 0.99 [0.85; 1.16] | 1.00 [0.99; 1.00] | 1.09 [0.98; 1.21] | 1.00 [1.00; 1.00] | 0.98 [0.85; 1.13] | 1.00 [0.99; 1.00] | 1.94 [1.31; 2.87] | 1.00 [1.00; 1.01] | 0.96 [0.57; 1.63] | 1.00 [0.98; 1.01] |
|  | **Obesity-rel. cancer** | 1.01 [0.93; 1.10] | 1.00 [1.00; 1.00] | 1.00 [0.89; 1.12] | 1.00 [1.00; 1.00] | 0.96 [0.84; 1.10] | 1.00 [1.00; 1.00] | 1.02 [0.86; 1.23] | 1.00 [1.00; 1.00] | 1.03 [0.93; 1.15] | 1.00 [1.00; 1.00] | 0.99 [0.85; 1.14] | 1.00 [0.99; 1.00] | 1.00 [0.92; 1.09] | 1.00 [1.00; 1.00] | 1.00 [0.89; 1.12] | 1.00 [1.00; 1.00] | 1.09 [0.70; 1.69] | 1.00 [1.00; 1.01] | 1.00 [0.56; 1.80] | 1.00 [0.98; 1.02] |
|  | **MI** | 1.05 [0.93; 1.19] | 1.01 [1.00; 1.01] | 0.96 [0.81; 1.13] | 1.00 [0.99; 1.00] | 0.97 [0.85; 1.11] | 1.00 [1.00; 1.00] | 0.95 [0.79; 1.14] | 1.00 [1.00; 1.00] | 1.54 [1.17; 2.03] | 1.02 [1.01; 1.03] | 0.96 [0.67; 1.38] | 0.99 [0.98; 1.01] | 1.04 [0.92; 1.17] | 1.00 [1.00; 1.01] | 0.96 [0.82; 1.14] | 1.00 [0.99; 1.00] | 1.60 [0.83; 3.10] | 1.02 [1.00; 1.04] | 0.83 [0.35; 1.99] | 0.99 [0.96; 1.02] |
|  | **Stroke** | 1.31 [1.17; 1.48] | 1.00 [1.00; 1.01] | 1.03 [0.87; 1.20] | 1.00 [1.00; 1.01] | 1.31 [1.12; 1.52] | 1.00 [1.00; 1.00] | 0.98 [0.80; 1.20] | 1.00 [1.00; 1.00] | 1.32 [1.09; 1.61] | 1.01 [1.00; 1.02] | 1.08 [0.83; 1.40] | 1.01 [1.00; 1.03] | 1.27 [1.12; 1.44] | 1.00 [1.00; 1.01] | 1.02 [0.86; 1.20] | 1.00 [1.00; 1.01] | 2.06 [1.37; 3.11] | 1.00 [0.99; 1.01] | 1.06 [0.62; 1.82] | 1.00 [0.99; 1.02] |
|  | **Diabetes** | 1.10 [1.03; 1.19] | 1.01 [1.01; 1.02] | 0.98 [0.89; 1.08] | 1.00 [0.99; 1.01] | 1.07 [0.97; 1.17] | 1.00 [1.00; 1.01] | 0.99 [0.87; 1.11] | 1.00 [0.99; 1.01] | 1.17 [1.04; 1.32] | 1.03 [1.02; 1.04] | 0.96 [0.82; 1.13] | 1.00 [0.98; 1.01] | 1.09 [1.01; 1.18] | 1.01 [1.01; 1.02] | 0.97 [0.88; 1.07] | 1.00 [0.99; 1.01] | 1.14 [0.74; 1.76] | 1.03 [1.01; 1.06] | 1.27 [0.76; 2.12] | 1.01 [0.97; 1.05] |
|  | **COPD** | 1.39 [1.28; 1.50] | 1.01 [1.00; 1.01] | 1.01 [0.91; 1.12] | 1.00 [1.00; 1.01] | 1.20 [1.06; 1.36] | 1.00 [1.00; 1.00] | 1.01 [0.86; 1.19] | 1.00 [1.00; 1.00] | 1.55 [1.40; 1.73] | 1.01 [1.01; 1.02] | 1.00 [0.87; 1.15] | 1.00 [0.99; 1.01] | 1.35 [1.24; 1.47] | 1.01 [1.00; 1.01] | 1.01 [0.90; 1.13] | 1.00 [1.00; 1.01] | 1.67 [1.29; 2.18] | 1.01 [1.00; 1.02] | 1.02 [0.72; 1.44] | 1.00 [0.98; 1.02] |
|  | **Anxiety** | 1.72 [1.63; 1.81] | 1.01 [1.00; 1.01] | 1.00 [0.93; 1.07] | 1.00 [1.00; 1.00] | 1.69 [1.55; 1.84] | 1.00 [1.00; 1.00] | 1.00 [0.89; 1.13] | 1.00 [1.00; 1.00] | 1.74 [1.63; 1.85] | 1.01 [1.01; 1.02] | 0.99 [0.91; 1.08] | 1.00 [0.99; 1.01] | 1.68 [1.58; 1.78] | 1.01 [1.00; 1.01] | 1.00 [0.92; 1.08] | 1.00 [1.00; 1.00] | 1.83 [1.65; 2.03] | 1.01 [1.00; 1.01] | 0.99 [0.87; 1.14] | 1.00 [0.99; 1.01] |
|  | **Depression** | 1.77 [1.70; 1.83] | 1.01 [1.01; 1.01] | 0.98 [0.94; 1.03] | 1.00 [0.99; 1.00] | 1.67 [1.57; 1.78] | 1.00 [1.00; 1.00] | 0.99 [0.91; 1.08] | 1.00 [1.00; 1.00] | 1.82 [1.74; 1.91] | 1.02 [1.01; 1.02] | 0.98 [0.92; 1.04] | 1.00 [0.99; 1.00] | 1.70 [1.63; 1.77] | 1.01 [1.00; 1.01] | 0.99 [0.93; 1.04] | 1.00 [0.99; 1.00] | 1.99 [1.84; 2.15] | 1.01 [1.00; 1.02] | 0.97 [0.88; 1.08] | 1.00 [0.99; 1.01] |

*The analyses compared participants who were overweight before the age of 30 and before diagnosis to those who were not. Regression and mediation models were adjusted for age at NAKO baseline examination, study center, and educational years. Sex was used as a covariate for the whole sample and the birth cohort subgroups. Smoking status was defined before disease diagnosis. CDE=controlled direct effect; PIE=pure natural indirect effect; INT_ref_=reference interaction; INT_med_=mediated interaction; rel.=related; MI=myocardial infarction; diabetes=type 2 diabetes; COPD=chronic bronchitis or chronic obstructive pulmonary disease; Anxiety=anxiety disorder or panic attacks; RR=risk ratio; 95%-CI=95% confidence interval*

**Table S11. Four-way decomposition** of the mediation analyses between childhood maltreatment and the presence of the diseases via **early-onset overweight during adulthood (18+)**.

|  |  | **Whole sample** | | | | **Men** | | | | **Women** | | | | **Older cohort (born <=1970)** | | | | **Younger cohort (born >1970)** | | | |
| --- | --- | --- | --- | --- | --- | --- | --- | --- | --- | --- | --- | --- | --- | --- | --- | --- | --- | --- | --- | --- | --- |
|  |  | **CDE** | **PIE** | **INT_re_** | **INT_med_** | **CDE** | **PIE** | **INT_re_** | **INT_med_** | **CDE** | **PIE** | **INT_re_** | **INT_med_** | **CDE** | **PIE** | **INT_re_** | **INT_med_** | **CDE** | **PIE** | **INT_re_** | **INT_med_** |
|  |  | **RR [95%-CI]** | **RR [95%-CI]** | **% [95%-CI]** | **RR [95%-CI]** | **RR [95%-CI]** | **RR [95%-CI]** | **% [95%-CI]** | **RR [95%-CI]** | **RR [95%-CI]** | **RR [95%-CI]** | **% [95%-CI]** | **RR [95%-CI]** | **RR [95%-CI]** | **RR [95%-CI]** | **% [95%-CI]** | **RR [95%-CI]** | **RR [95%-CI]** | **RR [95%-CI]** | **% [95%-CI]** | **RR [95%-CI]** |
| **Abuse** | **Any cancer** | 1.15 [1.07; 1.23] | 1.00 [1.00; 1.00] | 1.00 [0.91; 1.10] | 1.00 [0.99; 1.01] | 1.00 [0.88; 1.13] | 1.00 [1.00; 1.00] | 1.02 [0.86; 1.21] | 1.00 [1.00; 1.01] | 1.22 [1.12; 1.33] | 1.00 [1.00; 1.01] | 0.99 [0.88; 1.12] | 1.00 [0.99; 1.01] | 1.12 [1.04; 1.20] | 1.00 [1.00; 1.00] | 0.99 [0.90; 1.10] | 1.00 [0.99; 1.00] | 1.40 [1.09; 1.81] | 1.01 [0.99; 1.02] | 1.07 [0.76; 1.51] | 1.02 [0.99; 1.05] |
|  | **Smoking-rel. cancer** | 1.26 [1.14; 1.39] | 1.00 [1.00; 1.01] | 0.98 [0.86; 1.13] | 1.00 [0.99; 1.01] | 1.04 [0.82; 1.33] | 1.00 [1.00; 1.01] | 1.04 [0.74; 1.45] | 1.00 [0.99; 1.01] | 1.30 [1.17; 1.45] | 1.00 [1.00; 1.01] | 0.99 [0.85; 1.14] | 1.00 [0.99; 1.00] | 1.21 [1.09; 1.34] | 1.00 [1.00; 1.01] | 0.98 [0.85; 1.13] | 1.00 [0.99; 1.00] | 1.67 [1.18; 2.37] | 1.02 [0.99; 1.04] | 1.02 [0.63; 1.64] | 1.00 [0.96; 1.05] |
|  | **Obesity-rel. cancer** | 1.07 [0.98; 1.16] | 1.00 [1.00; 1.00] | 1.01 [0.90; 1.13] | 1.00 [0.99; 1.01] | 0.98 [0.85; 1.13] | 1.00 [1.00; 1.00] | 1.03 [0.85; 1.26] | 1.00 [1.00; 1.01] | 1.11 [1.00; 1.22] | 1.00 [1.00; 1.00] | 1.00 [0.87; 1.15] | 1.00 [0.99; 1.01] | 1.05 [0.97; 1.15] | 1.00 [1.00; 1.00] | 1.00 [0.89; 1.13] | 1.00 [0.99; 1.01] | 1.13 [0.79; 1.61] | 1.00 [0.98; 1.02] | 1.09 [0.67; 1.77] | 1.02 [0.98; 1.06] |
|  | **MI** | 1.28 [1.14; 1.45] | 1.01 [1.00; 1.01] | 0.96 [0.82; 1.14] | 0.99 [0.98; 1.00] | 1.24 [1.09; 1.43] | 1.00 [1.00; 1.01] | 0.96 [0.79; 1.16] | 1.00 [0.99; 1.00] | 1.47 [1.13; 1.93] | 1.02 [1.00; 1.04] | 0.97 [0.67; 1.41] | 0.99 [0.97; 1.02] | 1.29 [1.14; 1.45] | 1.01 [1.00; 1.01] | 0.96 [0.81; 1.14] | 0.99 [0.99; 1.00] | 0.94 [0.46; 1.91] | 1.02 [0.99; 1.05] | 1.28 [0.51; 3.20] | 1.05 [0.97; 1.13] |
|  | **Stroke** | 1.41 [1.25; 1.59] | 1.00 [1.00; 1.01] | 1.05 [0.89; 1.24] | 1.01 [1.00; 1.02] | 1.34 [1.14; 1.57] | 1.00 [1.00; 1.00] | 1.03 [0.83; 1.28] | 1.00 [1.00; 1.01] | 1.52 [1.27; 1.82] | 1.01 [1.00; 1.02] | 1.04 [0.81; 1.34] | 1.01 [1.00; 1.03] | 1.39 [1.23; 1.58] | 1.00 [1.00; 1.01] | 1.05 [0.88; 1.24] | 1.01 [1.00; 1.02] | 1.57 [1.05; 2.33] | 0.99 [0.97; 1.01] | 1.04 [0.60; 1.80] | 1.01 [0.97; 1.05] |
|  | **Diabetes** | 1.31 [1.22; 1.41] | 1.03 [1.02; 1.03] | 1.00 [0.91; 1.10] | 1.00 [0.99; 1.01] | 1.34 [1.22; 1.47] | 1.01 [1.00; 1.02] | 0.99 [0.87; 1.12] | 1.00 [0.99; 1.01] | 1.29 [1.15; 1.44] | 1.04 [1.03; 1.05] | 1.00 [0.85; 1.16] | 1.00 [0.98; 1.02] | 1.29 [1.20; 1.39] | 1.02 [1.01; 1.03] | 0.99 [0.90; 1.09] | 1.00 [0.99; 1.01] | 1.56 [1.12; 2.18] | 1.09 [1.06; 1.12] | 1.09 [0.72; 1.66] | 1.01 [0.96; 1.06] |
|  | **COPD** | 1.66 [1.54; 1.79] | 1.01 [1.01; 1.01] | 1.03 [0.93; 1.15] | 1.01 [1.00; 1.01] | 1.54 [1.37; 1.74] | 1.00 [1.00; 1.00] | 1.04 [0.89; 1.23] | 1.00 [1.00; 1.01] | 1.76 [1.59; 1.94] | 1.02 [1.01; 1.02] | 1.01 [0.88; 1.15] | 1.00 [0.99; 1.01] | 1.61 [1.49; 1.75] | 1.01 [1.00; 1.01] | 1.02 [0.91; 1.14] | 1.00 [1.00; 1.01] | 1.97 [1.58; 2.47] | 1.02 [1.00; 1.03] | 1.10 [0.81; 1.49] | 1.02 [0.99; 1.05] |
|  | **Anxiety** | 2.21 [2.11; 2.31] | 1.01 [1.00; 1.01] | 0.99 [0.93; 1.05] | 1.00 [0.99; 1.00] | 2.39 [2.21; 2.58] | 1.00 [1.00; 1.00] | 0.98 [0.88; 1.09] | 1.00 [0.99; 1.00] | 2.14 [2.02; 2.26] | 1.01 [1.00; 1.01] | 0.99 [0.92; 1.07] | 1.00 [0.99; 1.00] | 2.08 [1.97; 2.20] | 1.01 [1.00; 1.01] | 0.98 [0.91; 1.06] | 1.00 [0.99; 1.00] | 2.46 [2.27; 2.67] | 1.01 [1.00; 1.02] | 0.99 [0.88; 1.10] | 1.00 [0.99; 1.01] |
|  | **Depression** | 2.27 [2.19; 2.34] | 1.01 [1.01; 1.01] | 1.00 [0.95; 1.04] | 1.00 [1.00; 1.00] | 2.33 [2.20; 2.47] | 1.00 [1.00; 1.00] | 0.99 [0.92; 1.07] | 1.00 [1.00; 1.00] | 2.25 [2.16; 2.34] | 1.01 [1.01; 1.02] | 0.99 [0.94; 1.05] | 1.00 [0.99; 1.00] | 2.13 [2.05; 2.21] | 1.01 [1.00; 1.01] | 0.99 [0.94; 1.05] | 1.00 [0.99; 1.00] | 2.58 [2.42; 2.74] | 1.02 [1.01; 1.02] | 0.99 [0.91; 1.08] | 1.00 [0.99; 1.01] |
| **Neglect** | **Any cancer** | 1.07 [1.00; 1.15] | 1.00 [1.00; 1.00] | 1.01 [0.92; 1.11] | 1.00 [1.00; 1.00] | 1.01 [0.91; 1.13] | 1.00 [1.00; 1.00] | 1.03 [0.89; 1.20] | 1.00 [1.00; 1.00] | 1.11 [1.01; 1.21] | 1.00 [1.00; 1.00] | 0.99 [0.88; 1.12] | 1.00 [1.00; 1.00] | 1.05 [0.98; 1.13] | 1.00 [1.00; 1.00] | 1.01 [0.91; 1.11] | 1.00 [1.00; 1.00] | 1.43 [1.07; 1.91] | 1.00 [1.00; 1.01] | 1.06 [0.71; 1.57] | 1.00 [0.99; 1.01] |
|  | **Smoking-rel. cancer** | 1.11 [1.01; 1.23] | 1.00 [1.00; 1.00] | 1.01 [0.88; 1.16] | 1.00 [1.00; 1.00] | 1.13 [0.92; 1.40] | 1.00 [1.00; 1.00] | 1.00 [0.75; 1.34] | 1.00 [1.00; 1.00] | 1.10 [0.99; 1.24] | 1.00 [1.00; 1.00] | 1.01 [0.86; 1.18] | 1.00 [1.00; 1.00] | 1.07 [0.97; 1.19] | 1.00 [1.00; 1.00] | 1.00 [0.87; 1.15] | 1.00 [1.00; 1.00] | 1.90 [1.29; 2.79] | 1.00 [1.00; 1.01] | 1.05 [0.62; 1.79] | 1.00 [0.99; 1.02] |
|  | **Obesity-rel. cancer** | 0.99 [0.92; 1.08] | 1.00 [1.00; 1.00] | 1.02 [0.91; 1.15] | 1.00 [1.00; 1.00] | 0.96 [0.84; 1.09] | 1.00 [1.00; 1.00] | 1.04 [0.87; 1.24] | 1.00 [1.00; 1.00] | 1.01 [0.91; 1.13] | 1.00 [1.00; 1.00] | 1.00 [0.86; 1.16] | 1.00 [1.00; 1.00] | 0.99 [0.91; 1.07] | 1.00 [1.00; 1.00] | 1.02 [0.91; 1.14] | 1.00 [1.00; 1.00] | 1.11 [0.73; 1.70] | 1.00 [1.00; 1.00] | 1.04 [0.58; 1.86] | 1.00 [0.99; 1.01] |
|  | **MI** | 1.04 [0.93; 1.17] | 1.00 [1.00; 1.00] | 0.96 [0.82; 1.13] | 1.00 [1.00; 1.00] | 0.96 [0.84; 1.09] | 1.00 [1.00; 1.00] | 0.96 [0.80; 1.15] | 1.00 [1.00; 1.00] | 1.51 [1.16; 1.96] | 1.01 [1.00; 1.02] | 0.98 [0.68; 1.41] | 1.00 [0.98; 1.01] | 1.02 [0.91; 1.15] | 1.00 [1.00; 1.00] | 0.97 [0.83; 1.15] | 1.00 [1.00; 1.00] | 1.80 [0.99; 3.27] | 1.01 [0.99; 1.02] | 0.76 [0.33; 1.72] | 0.99 [0.97; 1.01] |
|  | **Stroke** | 1.31 [1.17; 1.47] | 1.00 [1.00; 1.00] | 0.99 [0.85; 1.17] | 1.00 [1.00; 1.00] | 1.27 [1.10; 1.47] | 1.00 [1.00; 1.00] | 1.00 [0.82; 1.23] | 1.00 [1.00; 1.00] | 1.38 [1.14; 1.66] | 1.01 [1.00; 1.01] | 0.98 [0.76; 1.27] | 1.00 [0.99; 1.01] | 1.27 [1.12; 1.43] | 1.00 [1.00; 1.00] | 0.99 [0.84; 1.17] | 1.00 [1.00; 1.00] | 2.08 [1.40; 3.10] | 1.00 [1.00; 1.00] | 1.01 [0.58; 1.75] | 1.00 [0.99; 1.01] |
|  | **Diabetes** | 1.10 [1.02; 1.18] | 1.00 [1.00; 1.01] | 0.98 [0.89; 1.08] | 1.00 [0.99; 1.01] | 1.08 [0.99; 1.18] | 1.00 [0.99; 1.00] | 0.96 [0.86; 1.09] | 1.00 [0.99; 1.01] | 1.13 [1.00; 1.26] | 1.02 [1.01; 1.03] | 1.00 [0.86; 1.17] | 1.00 [0.98; 1.02] | 1.09 [1.02; 1.17] | 1.00 [1.00; 1.01] | 0.97 [0.88; 1.07] | 1.00 [0.99; 1.01] | 1.06 [0.71; 1.58] | 1.01 [0.98; 1.05] | 1.26 [0.77; 2.07] | 1.00 [0.96; 1.06] |
|  | **COPD** | 1.38 [1.28; 1.49] | 1.00 [1.00; 1.00] | 1.02 [0.92; 1.13] | 1.00 [1.00; 1.01] | 1.19 [1.06; 1.34] | 1.00 [1.00; 1.00] | 1.02 [0.87; 1.20] | 1.00 [1.00; 1.00] | 1.56 [1.41; 1.73] | 1.01 [1.00; 1.01] | 1.01 [0.87; 1.16] | 1.00 [0.99; 1.01] | 1.36 [1.25; 1.47] | 1.00 [1.00; 1.00] | 1.01 [0.90; 1.12] | 1.00 [1.00; 1.00] | 1.66 [1.28; 2.16] | 1.00 [1.00; 1.01] | 1.12 [0.79; 1.58] | 1.00 [0.99; 1.02] |
|  | **Anxiety** | 1.72 [1.63; 1.80] | 1.00 [1.00; 1.00] | 1.00 [0.93; 1.07] | 1.00 [1.00; 1.00] | 1.68 [1.54; 1.83] | 1.00 [1.00; 1.00] | 1.00 [0.90; 1.13] | 1.00 [1.00; 1.00] | 1.74 [1.64; 1.85] | 1.00 [1.00; 1.01] | 1.00 [0.92; 1.09] | 1.00 [1.00; 1.00] | 1.67 [1.58; 1.77] | 1.00 [1.00; 1.00] | 1.01 [0.93; 1.09] | 1.00 [1.00; 1.00] | 1.84 [1.67; 2.04] | 1.00 [1.00; 1.01] | 0.98 [0.85; 1.12] | 1.00 [0.99; 1.00] |
|  | **Depression** | 1.75 [1.69; 1.81] | 1.00 [1.00; 1.00] | 1.00 [0.95; 1.05] | 1.00 [1.00; 1.00] | 1.64 [1.55; 1.75] | 1.00 [1.00; 1.00] | 1.01 [0.93; 1.10] | 1.00 [1.00; 1.00] | 1.81 [1.73; 1.90] | 1.01 [1.00; 1.01] | 0.99 [0.93; 1.05] | 1.00 [0.99; 1.00] | 1.69 [1.62; 1.76] | 1.00 [1.00; 1.00] | 1.00 [0.94; 1.05] | 1.00 [1.00; 1.00] | 1.98 [1.83; 2.13] | 1.00 [1.00; 1.01] | 1.00 [0.90; 1.10] | 1.00 [0.99; 1.01] |

*The analyses compared participants who were overweight during adulthood (18+) before diagnosis to those who were not, excluding participants who were solely overweight before diagnosis due to a higher weight compared to their peers at age 10. Regression and mediation models were adjusted for age at NAKO baseline examination, study center, and educational years. Sex was used as a covariate for the whole sample and the birth cohort subgroups. Smoking status was defined before disease diagnosis. CDE=controlled direct effect; PIE=pure natural indirect effect; INT_ref_=reference interaction; INT_med_=mediated interaction; rel.=related; MI=myocardial infarction; diabetes=type 2 diabetes; COPD=chronic bronchitis or chronic obstructive pulmonary disease; Anxiety=anxiety disorder or panic attacks; RR=risk ratio; 95%-CI=95% confidence interval*
